# When 20% Is Enough: Counterintuitive Contact Angle Maxima on Chemically Heterogeneous Hydrophobic/Hydrophilic Surfaces

**DOI:** 10.1021/acs.langmuir.6c00428

**Published:** 2026-05-28

**Authors:** Lorenzo Brugnati, Andrea Le Donne, Simone Meloni

**Affiliations:** Department of Chemical, Pharmaceutical and Agricultural Sciences, DOCPAS, Universitá di Ferrara, Via Luigi Borsari 46, 44121 Ferrara, Italy

## Abstract

Solid–liquid–gas three-phase systems are central to chemistry, biology, physics, engineering, and even botany. Representative examples range from gas-evolving reactions at (photo)­electrodes to hydrophobic interactions between proteins. Although these processes occur on the nanoscale, they are often interpreted using empirical principles extrapolated from macroscopic laws. Here we show, by means of atomistic simulations, that regularly distributed atomic- or molecular-scale chemical heterogeneities can give rise to counterintuitive behavior: the contact angle of an heterogeneous hydrophobic surface is maximized when the surface is decorated with a ∼20% atomic-level hydrophilic particle (with fixed interaction force). Similarly, a maximum in the contact angle is observed, at fixed hydrophilic particles geometry, when the difference in interaction force between the hydrophobic and hydrophilic spots is Δ ∼ 40%. We demonstrate that such atomistic-level heterogeneities can *pin* the solid–liquid–gas contact line. These findings have several implications. First, they rationalize the high hydrophobicity of materials that nonetheless contain a significant fraction of hydrophilic sites, Cu_2_(tebpz), a metal–organic framework whose pores host Cu nodes, being a notable example. Second, if our results are experimentally confirmed for more general, nonregularly patterned surfaces, they will add a new evidence for the need of overcome the *asymptotic homogenization*, widely used for describe heterogeneous surfaces from individual component, such as protein surfaces with amino-acid hydrophobicity scales or nanoporous materials.

## Graphical abstract

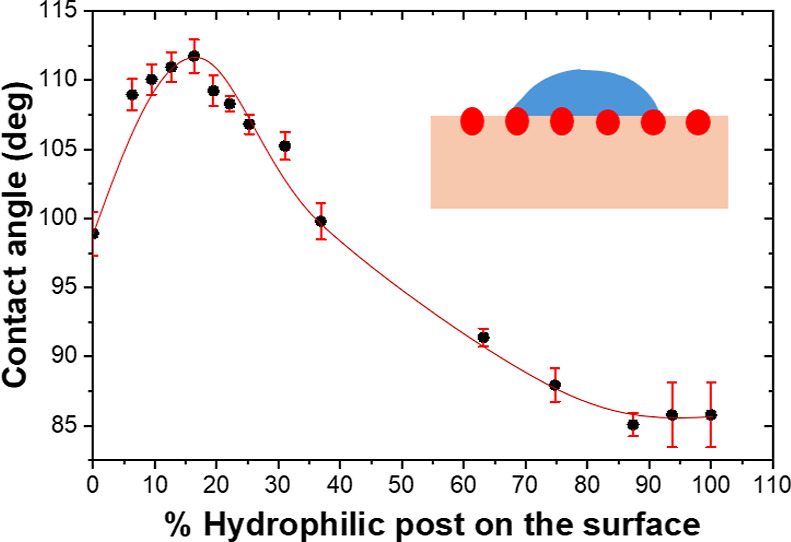

## 1 Introduction

Interface systems between solids and fluids (liquids or gases) play a crucial role in many fields, ranging from chemistry^1^ to physics,^2^ from biology^3^ to engineering.^4,5^ The phenomena investigated in this work concern the hydrophobicity (more generally, lyophobicity) of materials. Hydrophobicity of materials, and the associated *hydrophobic interaction*, is responsible for the strong adhesion between solid hydrophobic surfaces immersed in water,^6^ micelle formation,^7^ and protein folding,^8,9^ just to name a few examples that have attracted significant interest from both the experimental and theoretical communities. More recently, fluids confined within hydrophobic meso- (≥2 nm) and microporous (<2 nm) systems^10^ have been extensively investigated because of their peculiar properties, the differences between bulk and confined water in contact with a solid surface, and the possible technological applications of these systems.

Hydrophobicity (or hydrophilicity) of a material is conventionally measured by its contact angle θ_*Y*_ with a water droplet, the angle between the tangent to the surface of the droplet and the solid surface at their contact point (Figure 1). Following Young (see Section 2, namely eq 1), the contact angle of a droplet in equilibrium on a surface is a convenient measure of the relative strengths of the solid/liquid and solid/gas (or solid/vapor) energies, compared with the liquid surface tension. This equation, however, concerns the macroscopic picture of the system, where the solid, liquid and gas are assumed to be continuum. In the Young equation, the solid is assumed to be *smooth* and chemically uniform on the macroscopic scale. Real surfaces, however, typically deviate from this ideal picture. Concerning physical heterogeneity, i.e., surface roughness, Wenzel^11^ and Cassie and Baxter^12^ developed models connecting the Young contact angle and the physical surface characteristics to the so-called apparent contact angle, the angle measured on a real, nonsmooth surface when the liquid wets the cavities and when it is suspended over the asperities, respectively. More general relations for surfaces combining physical and chemical heterogeneity have been proposed (see, e.g., ref 13). Still, these relations are based on a macroscopic picture of the multiphase system, where its energetics is expressed in terms of surface tensions, with the possible inclusion of a line-tension term, as we discuss more specifically below in Section 2.

**Figure 1:**
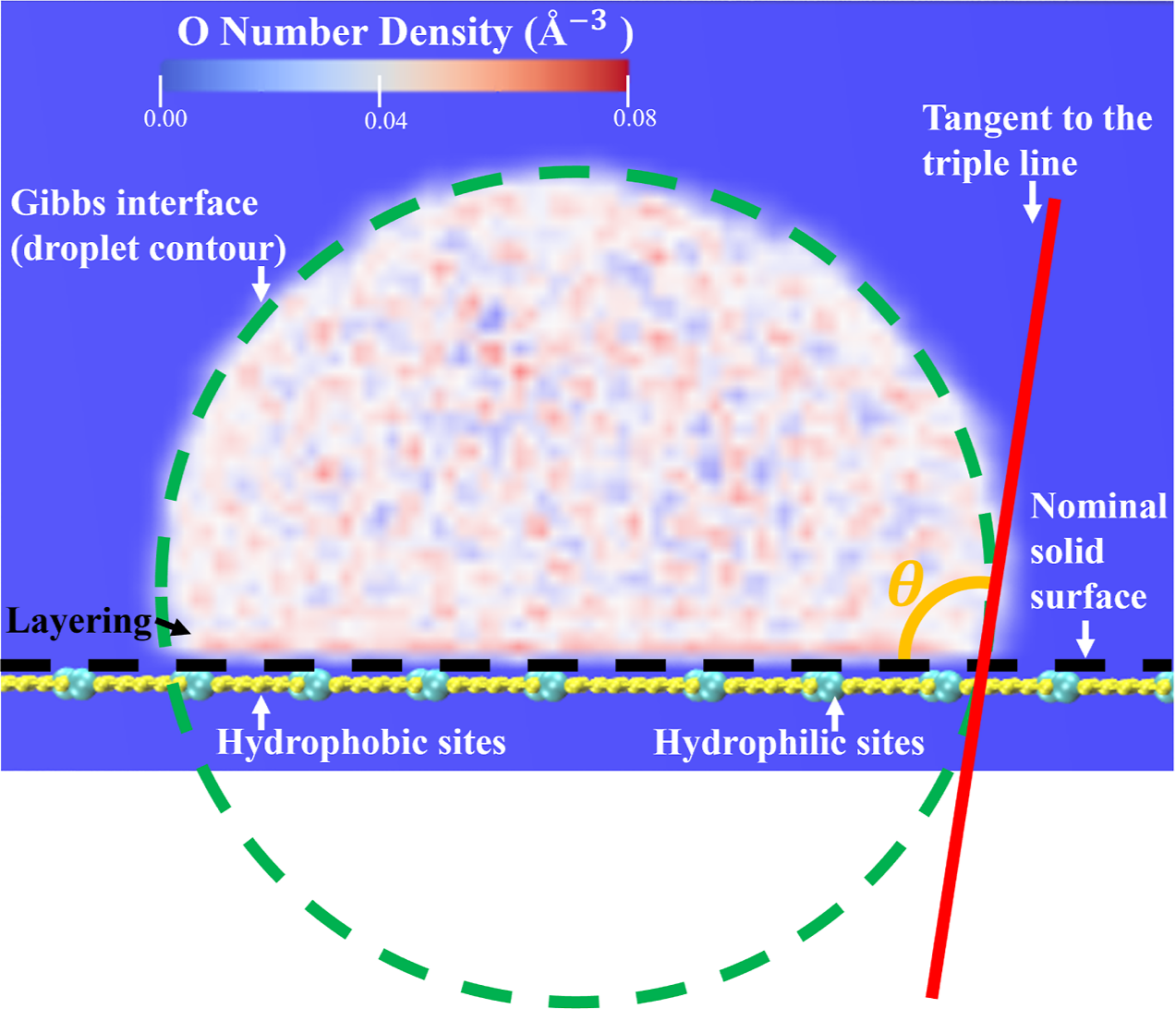
Schematic illustration of the contact angle determination method used in this work. The geometrical approach employed here is computational counterpart of the experimental one. The water density distribution is shown: blue areas indicate regions devoid of water molecules, red areas indicate high water density. Some inhomoneneities are observed in the nanodroplet due to finiteness of simulation time. The typical layering pattern, a higher water density region near the solid surface, is observed (see Section 3). From this distribution the water droplet contour, defined as the Gibbs dividing surface where the water density equals one-half of the bulk value, is extracted. This contour is then fitted with a circle (green dotted line). The nominal solid surface is located 3 Å outermost atoms (hydrophilic atoms are represented as full spheres, while hydrophobic atoms are shown in yellow). The triple-phase contact point is determined at the intersection between the fitted circle and the nominal solid surface. The contact angle is defined by the tangent to the circle at this intersection point (red line).

The relation between microscopic characteristics, e.g., the chemical composition of a surface, and macroscopic properties, such as the contact angle and the strength of hydrophobic interactions,^14^ has attracted attention. The design of surfaces with tailored properties, on the one hand, and the understanding of the origin of the characteristics and functionalities of existing surfaces, e.g., biological ones, on the other, requires the identification of laws connecting the micro- and macroscopic descriptions of solid/fluid interactions. In this regards, the theoretical tool connecting the two worlds is asymptotic homogenization,^14–16^ where the average of microscopic interactions is used to represent macroscopic properties.

Giovambattista, Debenedetti, and Rossky investigated the effect of nanoscale chemical heterogeneity in a number of pioneering works (see, e.g., refs 17 and 18 ). They considered heterogeneous *mesoscopic* domains, namely, square plates consisting of a ∼1 nm hydrophilic core and a ∼1 nm hydrophobic border, and vice versa. Together with other studies (e.g., ref 19) they highlight the importance of atomistic-level heterogeneity for the hydrophobicity/hydrophilicity of a material and reveal the influence that neighboring domains of differing hydrophilicity/hydrophobicity exert on each other.

An intriguing case is that of confined heterogeneous surfaces, e.g., the inner walls of some zeolites (see, e.g., refs 20 and 21 ) or metal–organic frameworks (MOFs) such as HKUST-1 ^22^ or Cu_2_(tebpz).^23–25^ These materials are important both for their fundamental properties and for their technological applications.^26–28^ Regarding the hydrophilic/hydrophobic nature of porous media, applications in energy storage, energy dissipation, and energy damping have emerged over the last 20 years.^2,29,30^ Proteins, with surfaces alternating more and less hydrophilic or hydrophobic peptides, may likewise represent systems whose dynamics (e.g., folding) could be driven by unexpected effects of atomistically heterogeneous surfaces, as anticipated in recent works.^31,32^ In these works the authors demonstrated the relation between the heterogeneity of surfaces with *wettability* and double-layer formation, and the origin of this phenomenology. For example, Rego et al.^33^ shows that, contrary to intuition, an excess of polar groups can sometimes lead to a more hydrophobic environment.

To illustrate the puzzling physics of atomistically heterogeneous surfaces, consider Cu_2_(tebpz). This MOF is highly hydrophobic, with a ∼130° contact angle and an intrusion pressure, the hydrostatic pressure one must apply on a liquid suspension of water and the MOF to intrude the pores of the material, of ∼25 MPa at ∼300 K, which grows to ∼35 MPa at 380 K. However, in apparent contradiction, before intrusion Cu_2_(tebpz) contains a large amount of water in the form of vapor pressure, estimated at several MPa.^25^ How can the high contact angle and high intrusion pressure of Cu_2_(tebpz) be reconciled with its high water-vapor content? Water is attracted to copper atoms present in the MOF, but how does this align with the overall high hydrophobicity of the material? Finally, how can an internal surface that hosts two hydrophilic sites (copper) per hydrophobic molecule (tebpz) still be highly hydrophobic (∼130°)?

Intuition suggests that adding more hydrophilic atoms or molecules makes a system more hydrophilic (or less hydrophobic), and vice versa. Here, exploiting the flexibility of atomistic simulations, we show that this is not necessarily the case. In particular, we show that a heterogeneous Lennard-Jonesium surface composed of hydrophilic and hydrophobic particles, distributed according to the topology and geometry of copper atoms and tebpz linkers in the Cu_2_(tebpz) MOF, presents a maximum in the contact angle when ∼20% of the surface particles are hydrophilic or, at a fixed geometry, the interaction force between the hydrophilic “spots” and the water molecules is ∼+40% stronger than with *hydrophobic* ones. To the best of our knowledge, this is the first time it has been shown that the contact angle of a surface may present a maximum as the number of atomic- or molecular-sized hydrophilic spots increases. If confirmed experimentally, and upon a broader exploration of the dependence of this phenomenology with the topology of hydrophilic spots, present findings reveal unexpected physics that may lead to novel design principles for surfaces with targeted properties.

The article is organized as follows: Section 2 outlines the theoretical framework and the computational methods employed. Section 3 presents and discusses the results. Finally, Section 4 provides some concluding remarks.

## 2 Theory and Computational Methods

### 2.1 Contact Angle

The hydrophobic or hydrophilic nature of a material is typically measured by the contact angle formed by a liquid droplet deposited on its surface. The contact angle, θ_*Y*_, is defined as the angle formed by the tangent to the droplet surface at its contact point with the solid (Figure 1). Young showed that the contact angle of an equilibrium droplet, a geometric property of the solid/droplet system, is related to thermodynamic quantities, namely the surface tensions of the three phase pairs: solid–liquid, solid–gas, and gas–liquid.^34^ The Young equation reads

1 $$\cos\left(\theta_{Y}\right)=\frac{\gamma_{SG}-\gamma_{SL}}{\gamma_{\mathrm{LG}}}$$

where γ_*XY*_ is the surface tension between phases *X* and *Y* and, in particular, γ_SL_, γ_SG_, and γ_LG_ denote the solid–liquid, solid–gas, and liquid–gas surface tensions, respectively. If γ_SL_ > γ_SG_, it is energetically more expensive for the solid to be in contact with the liquid than with air; in this case, θ_*Y*_ > 90° and the system is termed hydrophobic. Conversely, when γ_SL_ < γ_SG_, θ_*Y*_ < 90° and the system is termed hydrophilic. Of course, the distinction between hydrophobic and hydrophilic surfaces based on the contact angle is arbitrary; it simply reflects the energetic *preference* of a material to form an interface with water or air.

In the Young equation, the solid–liquid and solid–gas surface-tension terms refer to a *smooth* and chemically uniform surface. Real surfaces, however, typically deviate from this ideal model. For physical heterogeneities, surface roughness or texture, Wenzel^11^ and Cassie and Baxter^12^ developed equations that connect the so-called *apparent* contact angle (the experimentally measured angle) to the Young contact angle and the physical surface characteristics. With respect to chemical heterogeneity, eq 1 is justified by asymptotic homogenization,^14–16^ which uses a macro- or mesoscale average of microscopic solid/liquid interactions.

Intuitively, one expects that the contact angle of a surface containing domains of higher and lower hydrophobicity/hydrophilicity would lie between the extreme values. However, when the domains with stronger and weaker attraction are on the same scale of the size of the liquid (droplet) or gas (bubble) phases, such averaging is less obvious (see Section 3.1.2). This is the effect we investigate in this work. In particular, we compute the contact angle of surfaces as a function of the concentration of hydrophilic and hydrophobic particles and of the strength of their interaction with water.

The contact angle can be determined from atomistic simulations using several approaches. Caddeo et al.^35^ and Le Donne et al.^36^ employed an approach based on the Young–Dupré equation, an alternative form of eq 1 written in terms of the liquid surface tension γ_LG_ and the adhesion free energy, Δ*G*_adh_, between the solid and the liquid. This approach conveniently sidesteps the droplet-size issue present in the *geometrical* approach^37–39^ discussed below.

Le Donne et al. showed that entropic contributions to the adhesion free energy are typically large, so Δ*G*_adh_ cannot be approximated by the corresponding enthalpy.^36^ Reliable results therefore require computing the free-energy difference between separated and contacted liquid and solid, which entails calculating and integrating a suitable mean force, a computationally expensive procedure unsuited to exploring chemical heterogeneity over a broad composition range. Consequently, we adopt the geometrical approach widely used in the literature (see, e.g., ref 40). A droplet is deposited on the solid surface, and its contact angle is determined from the liquid density field, as explained in detail below. The dependence of the contact angle on droplet size is minimized by employing a cylindrical droplet (Figure 2,a). The effect of the size of the droplet on the contact angle is discussed in the following section.

**Figure 2:**
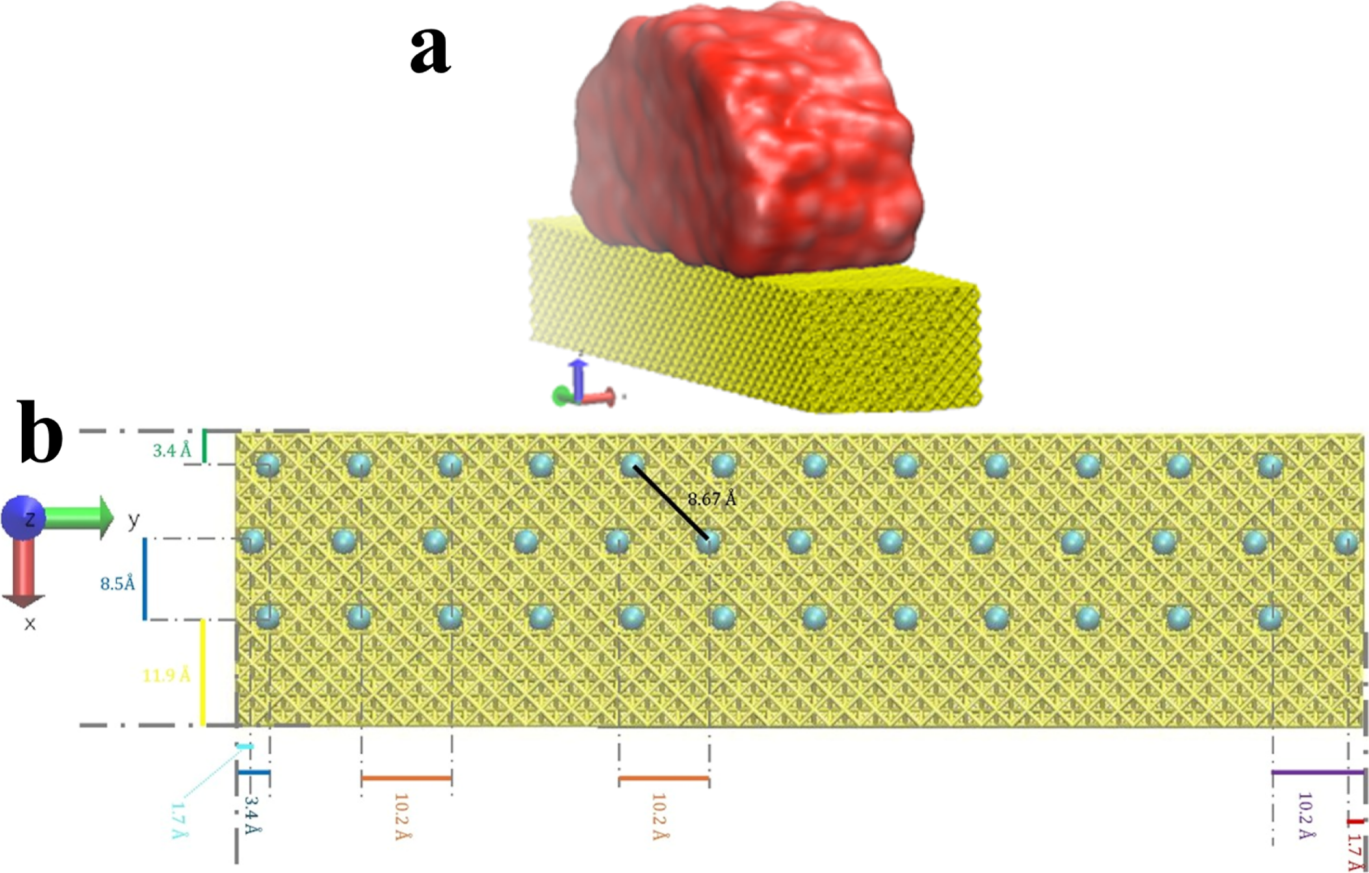
(a) Snapshot of the simulated system. In red the external instantaneous surface of the water droplet consisting of 3000 molecules is shown. In yellow the hydrophobic material is depicted, comprising the (100) surface of an FCC crystal of Lennard-Jones particles. The slab contains approximately 20,000 atoms. Chemically heterogeneous samples were obtained by replacing a suitable number of particles in the top layer with others that interact with water more strongly. Panel (b) shows one of the patterned surface used in this work, denominated “3 lines” patterned surface in the following. This surface consists of 3 lines of hydrophilic spots distributed consistently with hydrophilic copper atoms in the Cu_2_(tebpz) MOF. The complete set of patterned surfaces considered in this work are presented in Supporting Information Figures SI-1–SI-3.

In practice, the geometrical approach proceeds as follows (see Figure 1). From the molecular-dynamics trajectory, one extracts the discretized two-dimensional water-density field of the droplet on the surface. The droplet’s contour is defined as the set of points where the water density equals one-half the bulk value (the Gibbs interface). These points are fitted by a circle whose parameters are the two-dimensional coordinates of its center and its radius. The tangent to this circle at its intersection with the solid surface defines the contact angle. On the atomistic scale, the contact point between the circle and the solid is not uniquely defined because it depends on where the top surface of the solid is placed. For example, one can place this surface at the positions of the atoms in the top layer or one atomic radius above that. Although the choise is arbitrary, reasonable variation of the nominal solid surface slightly modify the absolute value of the contact angle but not the overall trend, as is possible to see in Supporting Information Figures SI-8 and SI-9. Here, the nominal solid surface is set 3 Å above the positions of the atoms in the top layer (Figure 1).

This approach, and indeed the concept of a contact angle itself, is meaningful only when the droplet is sufficiently large for bulk-water properties to be recovered in its interior. In our case, the droplet radius is about 20 times the molecular radius of water, implying an analogous ratio with the liquid–gas interfacial thickness. The droplets considered here are therefore large enough to exhibit bulk-water behavior in their central region, as confirmed by the density field shown in Figure 7. Moreover, as demonstrated in Figures 5, 9, along the direction orthogonal to the surface, after an initial layering region, bulk-like conditions are also recovered. This further supports that, not only in the lateral plane but also in the normal direction, the use of the contact angle is well justified.

### 2.2 Size Effect

The dependence of the contact angle of a droplet on its size is known.^39,41^ For droplets in their equilibrium configuration, this size effect can be rationalized in terms of three phase contact line effects,^42,43^ hereinafter referred to as contact line for brevity

2 $$\cos\left(\theta \right)=\cos\left(\theta_{Y}\right)+\frac{\tau }{\gamma_{\mathrm{LG}}}R_{b}^{-1}$$

where θ is the apparent contact angle, θ_*Y*_ is the Young contact angle, τ is the line tension, *R*_b_ is the base radius of the droplet and γ_LG_ is the liquid gas surface tension.

Equation 2 shows that the contact angle intrinsically depends on droplet size through *R*_b_, with the ratio τ/γ_LG_ determining the magnitude of this effect. For cylindrical droplets, *R*_b_ is independent of droplet size, and the curvature of the droplet base remains constant (indeed, infinite); therefore, any size dependence can only originate from γ_LG_. The size dependence of γ_LG_ is governed by the Tolman length δ. For water, δ = 0.05 nm,^44^ which is more than 2 orders of magnitude smaller than the droplet size considered in this work (∼6.5–7 nm). Therefore, in the absence of pinning, the contact angle of a cylindrical droplet is expected to depend only weakly on droplet size. Indeed, cylindrical droplets are widely used in computational studies of solid–liquid–gas interfaces,^37,45^ and Weijs et al.^42^ reported that the size dependence of the contact angle is negligible for cylindrical droplets larger than ∼5 nm.

Conversely, an observable dependence of the contact angle on droplet size may indicate pinning at the contact line.

### 2.3 Computational Setup

Classical molecular-dynamics (MD) simulations were performed with the LAMMPS code.^46^ Water was modeled using the TIP4P/2005 potential,^47^ in which each water molecule comprises four sites: two hydrogen atoms, an oxygen atom, and a massless site *M* lying ∼0.15 Å away from the oxygen atom toward the hydrogens. Fixed point charges reside on the hydrogens and on the *M* site. Van der Waals interactions between water molecules and between water and the solid are described by the Lennard-Jones potential; the oxygen atom is the only Lennard-Jones center in the molecule. TIP4P/2005 model provides, among other properties, an accurate surface tension for water (69.3 mN m^–1^ versus the experimental 71.73 mN m^–1^),^48^ very important for this research.

For the solid we considered both chemically homogeneous and heterogeneous samples. In all cases, solid particles interact with water via a Lennard-Jones potential^49^

3 $$V_{LS}^{\alpha }\left(r\right)=4\varepsilon_{LS}^{\alpha }\left[{\left(\frac{\sigma_{LS}^{\alpha }}{r}\right)}^{12}-{\left(\frac{\sigma_{LS}^{\alpha }}{r}\right)}^{6}\right]$$

where *r* is the distance between a solid particle and the oxygen atom of a water molecule. The parameters ε_LS_^α^ and σ_LS_^α^ are the depth and the zero-crossing distance of the potential between water and solid particles of type α. For homogeneous samples there is only one parameter set, so the index α is superfluous. For heterogeneous samples, two solid species are present, each with its own ε_LS_^α^ but same σ_LS_^α^.

Two families of heterogeneous systems were investigated. In the first, a fixed number of hydrophilic particles are regularly distributed on an otherwise hydrophobic surface (see the Supporting Information Figures SI-1–SI-3). The particles are *hydrophilic* in the sense that they interact more strongly with water (larger value of ε_LS_^α^). Here, the hydrophilicity of the spots is tuned by increasing ε_LS_^α^, and the resulting change in the contact angle is measured. In the second family, the number of regularly distributed hydrophilic spots is varied while their interaction strength (i.e., ε_LS_^α^) is kept constant; the contact angle is then measured as a function of the fraction of hydrophilic spots on the surface. Figure 3 shows the relationship between ε_LS_^α^ and θ_*Y*_ for a chemically uniform surface at 300 K.

**Figure 3:**
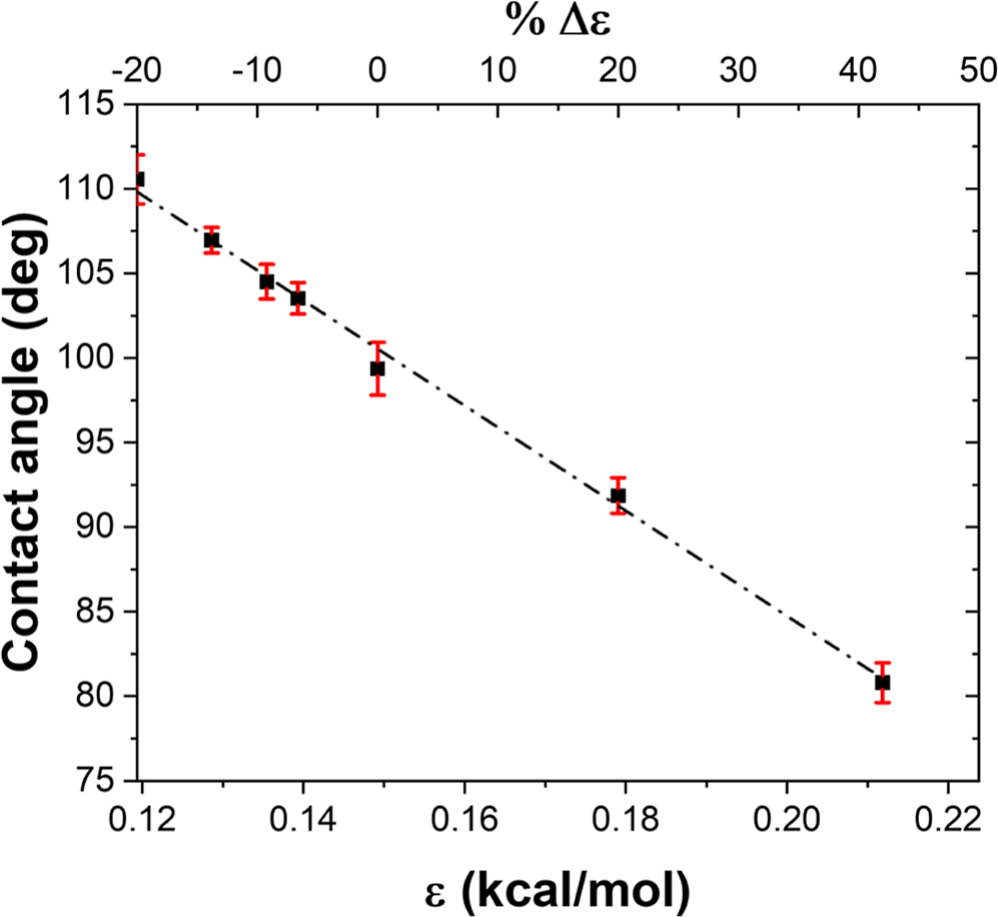
Dependence of the contact angle on the strength of the solid–liquid interaction for a surface with homogeneous chemistry. The percentage variations on the top *x* axis are arbitrarily referenced to 0.14920 kcal mol^–1^ (this value is used in the following sections for the hydrophobic part of the material). This representation helps to build an intuitive idea of the change in solid–liquid strength required to modify the contact angle by a desired amount. The method used to compute the error on the contact angle for data in this and following charts is discussed in the Supporting Information Section 1.

Our MD systems contain ∼20,000 solid particles in the slab and 3000 water molecules in the droplet, corresponding to a droplet diameter of ∼6.5–7 nm. Simulations were carried out in the *NVT* ensemble (constant number of particles, volume, and temperature) using the Nosé–Hoover chains thermostat^50^ at 300 K. Each simulation was initialized with the water droplet positioned 6 nm above the solid particle. In addition to their thermal motion, all atoms were assigned a small drift velocity directed toward the surface. This initial drift imparts sufficient kinetic energy for the droplet to overcome minor energy barriers and avoid arrest in metastable states near the initial impact position. Once contact was established, the system was equilibrated for 15 ns. Data from the final 2.5 ns were used for analysis. Error bars were estimated using the jackknife method, as described in detail in Section 1 of the Supporting Information. The consistency of the qualitative trends across different numbers of hydrophilic sites, as well as across different interaction strengths at fixed site number, supports the conclusion that the reported behavior is not an artifact of the initialization procedure. As illustrated in Figure 2a, sufficient space is left in the *z* direction, the direction orthogonal to the surface, to prevent any spurious interaction between water and material through the periodic boundary conditions.

## 3 Results and Discussion

In the following section we consider two cases of heterogeneous surfaces:

- surfaces containing a fixed number of hydrophilic particles, regularly distributed on an otherwise hydrophobic surface, while the strength of the hydrophilic *spots* is varied;
- surfaces containing a variable number of hydrophilic particles of fixed strength, regularly distributed on an otherwise hydrophobic surface.

In both cases, the arrangement of the spots is consistent with the positions of Cu atoms in Cu_2_(tebpz)^23,25,51^ (see Figure SI-1).

We start our analysis from the reference case of a chemically homogeneous surface, determining the trend of θ_*Y*_ vs ε. As expected, θ_*Y*_ diminishes with increasing ε, confirming the intuitive argument that for chemically homogeneous surfaces increasing the strength of the interaction between solid particles and the liquid makes the surface more hydrophilic (Figure 3).

### 3.1 Contact Angle vs Strength of the Water/Particle Interaction of Hydrophilic Spots

In this section we consider three cases: solids with hydrophilic particles arranged in 2, 3, and 4 rows (see Figure SI-1). The case with 4 rows of hydrophilic spots corresponds to the Cu patterning of Cu_2_(tebpz). For all cases we determined the dependence of θ_*Y*_ on ε, the strength of the interaction between the hydrophilic particles and water. The strength of the same interaction with hydrophobic particles is kept constant at ε = 0.14920 kcal mol^–1^. Results obtained for the three geometries are qualitatively analogous; hence, in the main text we present and discuss the results obtained for the case with three rows of hydrophilic spots. The cases with 2 and 4 rows are reported in the Supporting Information (Figures from SI-4–SI-7).

The results challenge intuition: θ_*Y*_ increases by ∼15° when the attraction between the spots and water is strengthened by ∼40% with respect to the initial all-hydrophobic surface (Figure 4). For even stronger interactions between hydrophilic spots and water, the contact angle decreases. In other words, the contact angle exhibits a maximum when the surface is decorated with particles whose interaction with water is about 40% stronger than the reference value.

**Figure 4:**
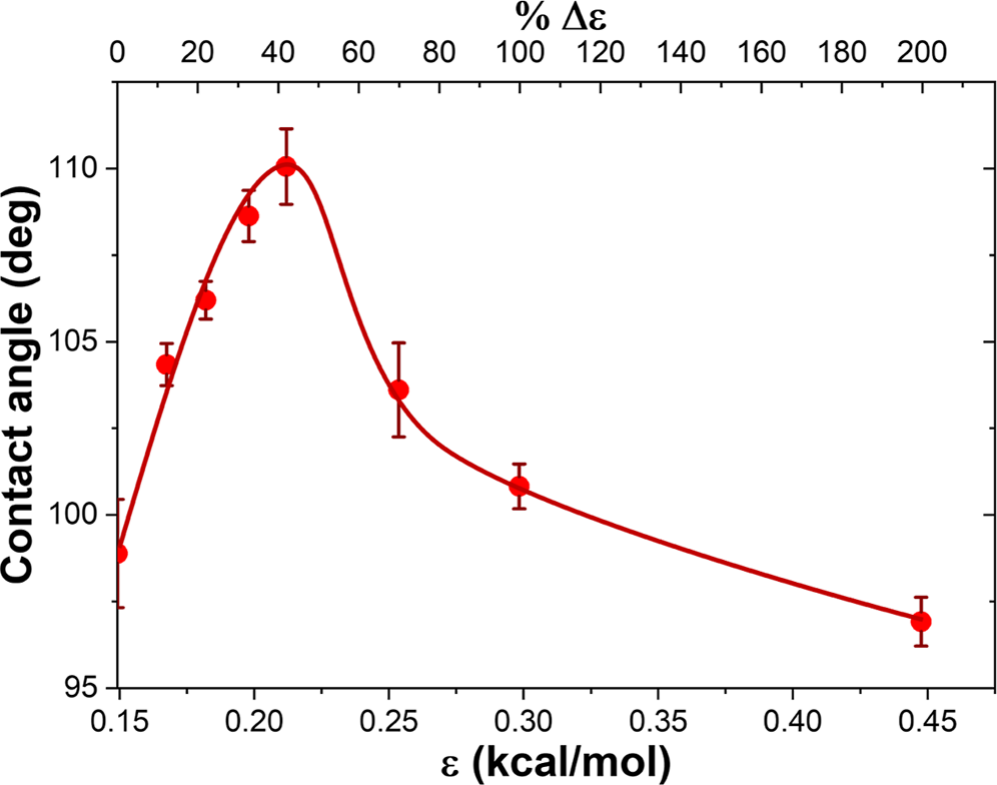
Contact angle vs the strength of the water/surface interaction for the case of “3 lines” of hydrophilic spots. The percentage variations (top *x* axis) refer to ε = 0.14920 kcal mol^–1^. The red line in the chart is added only as a visual guide.

Contrary to the results of Weijs et al.,^42^ who reported that, in the absence of pinning, the contact angle of cylindrical droplets depends only negligibly on droplet size over the range considered here, we observe a noticeable size effect (Figure SI-10). Nevertheless, while droplet size changes the absolute value of the contact angle, the unusual nonmonotonic trend persists for all nanodroplets considered in this work. In particular, the contact angle displays a maximum at Δε ∼ +42% in every case. Notice that our results do not conflict with those of ref 42, because, as discussed below, pinning of the contact line cannot be ruled out in the present system.

To rationalize the unexpected trend of results of Figure 4, we considered two hypotheses based on the established understanding of how the contact angle depends on the properties of the solid surface on which the droplet is deposited:

1. the heterogeneous surface produces an anomalous effective solid–liquid interaction strength;
2. atomic-scale chemical heterogeneity generates strong contact line pinning that initially increases the contact angle. At sufficiently strong water attraction from hydrophilic spots, however, wettability dominates over pinning and the contact angle decreases.

#### 3.1.1 Anomalous Effective Solid–Liquid Interaction Strength Hypothesis

To test the first hypothesis, we computed the liquid number-density profile as a function of the distance from the surface (Figure 5). The profile shows the typical layered structure observed for liquids in contact with solids,^52–54^ characterized by maxima and minima of progressively decreasing intensity. The insets show enlarged views of these features. Since (in absence of pinning) the amplitude of the density oscillations reflects the overall attractive or repulsive character of the solid–liquid interaction,^55^ hypothesis (1) would imply a correlation between the amplitudes of the maxima and minima and the strength of the interaction between water and the hydrophilic sites, in a manner consistent with the observed trend in contact angle. Instead, the height of the maxima increases with the strength of the interaction between the hydrophilic particles and water. In other words, we observe a regular trend of the density profile with the strength of the interaction of water with the surface particles (Figure 5). In conclusion, we observe no correlation between the trend of the density profile with the strength of the liquid–solid interaction and the nonmonotonic trend of the contact angle (Figure 4). This led us to exclude hypothesis (1).

**Figure 5:**
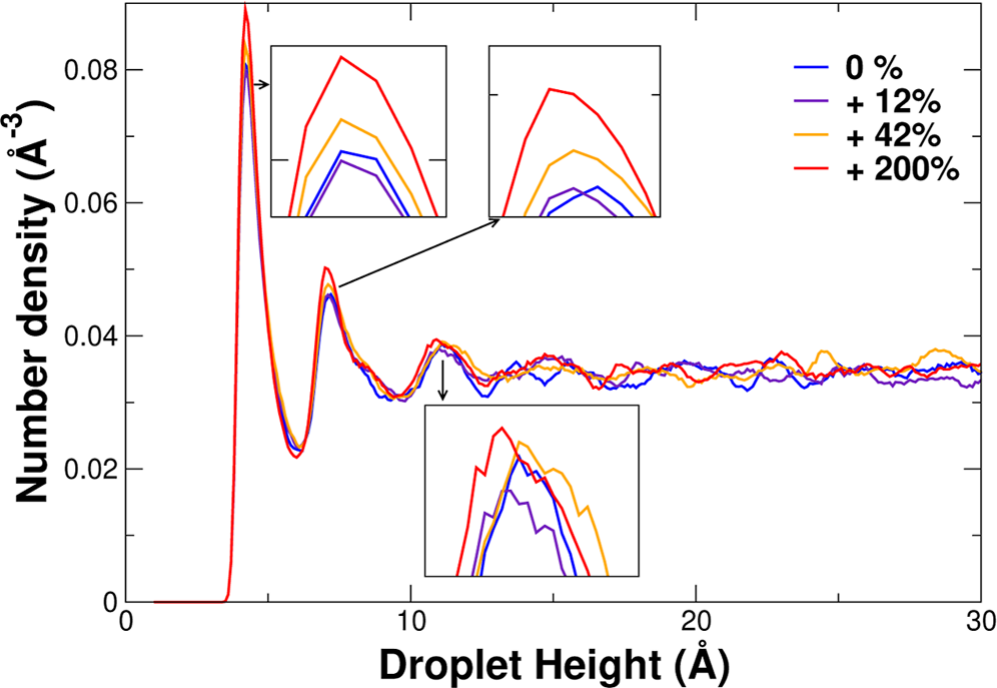
Profile of the water density field ρ­(*x*) as a function of the distance from the surface. The ρ­(*x*) profile is computed for a purely hydrophobic surface (0% in the legend, blue profile) and for heterogeneous surfaces with hydrophilic spots of different interaction strengths with water. The profile shows the typical layering effect—maxima and minima of the liquid density—observed when water is brought in contact with a surface. The maxima display an increasing height with increasing interaction strength of the hydrophilic spots with water.

Remarkably, the present results, particularly the regular trend of the density peaks with increasing particle hydrophobicity, are somewhat at odds with those reported in ref 56. That work shows that amphiphilic bilayers systems made with organic chains terminated with hydrophilic OH and CHCN groups exhibit a reduced liquid density in the interfacial layers compared with analogous hydrophobically terminated systems (e.g., –CF_3_, –CH_3_, –OCH_3_), whereas the more hydrophilic CONH_2_ termination yields a first water-layer density comparable to that observed for hydrophobically terminated chains.

The above observations suggest that the unexpected trend of the contact angle with the strength of the hydrophilic particles may be due to hypothesis two, i.e., by a combination of stronger adhesion of water to the surface—arising from the increased ε of the hydrophilic particles—together with pinning of the three phase contact line. Beyond a threshold ε, the effect of adhesion overcomes the effect of pinning and the contact angle decreases. A problem with this hypothesis is that, so far, pinning of the contact line by atomistic-level heterogeneities has not been investigated. We undertake this investigation here, and the results are presented and discussed in the next section.

#### 3.1.2 Three-Phase Contact Line Pinning on an Atomistically Smooth Surface Hypothesis

Pinning and the ensuing contact angle hysteresis are due to surface chemical and physical heterogeneities.^57,58^

Pinning originates from metastable states on the droplet free-energy landscape associated with the evolution of collective degrees of freedom,^59^ rather than with the behavior of individual water molecules. For example, Giacomello et al. showed that a protuberance only 1 nm thick on an otherwise chemically and physically homogeneous surface is sufficient to induce pinning.^60^ Similarly, sharp edges on chemically and physically homogeneous surfaces also give rise to pinning,^61^ which ultimately underpins Gibbs’ criterion for liquid advancement beyond an edge.^62^

For chemical heterogeneities, it has been shown that mesoscopic domains of several tens of nanometers give rise to pinning.^63,64^ Some authors have reported a dependence of the pinning capability on the size of the chemical heterogeneities,^65^ but, to the best of our knowledge, only continuum one-dimensional heterogeneities (stripes of materials with different characteristics) have been considered. Do strictly atomistic (subnanometric) heterogeneities, smaller than those studied previously, lead to contact line pinning and, hence, contact angle hysteresis?

An affirmative answer to this question would pose an additional challenge to homogenization theory, which seeks to connect microscopic and mesoscopic/macroscopic descriptions of matter in models of solid/liquid/gas interfacial systems. Specifically, the atomic nature of the system could not be described in terms of its locally asymptotically homogeneous counterpart, which would instead predict no hysteresis. Such atomic-scale heterogeneities are unavoidable even on ideal surfaces of multicomponent materials, including the surfaces of single-crystal solids and the inner walls of porous materials. This result is particularly relevant for porous materials such as MOFs and zeolites, which often feature regular patterns of hydrophilic sites and support solid/liquid/gas interfaces on the length scale considered in this work. One example is the metal–organic framework Cu_2_(tebpz), whose surface chemistry inspired the model surfaces studied here. Cu_2_(tebpz) contains cylindrical nanometric cavities with regularly patterned hydrophilic sites on otherwise hydrophobic inner walls, and shows water-intrusion behavior that challenges intuition.

Here, we do not analyze in detail the possible origin of this phenomenon; we limit our attention to investigating and reporting the phenomenology for the systems considered in the previous section, i.e., for a system presenting ordered set of atomic-size chemical heterogeneities.

We investigate triple-line pinning by determining the difference, if any, between the left and right contact angles, Δθ_hist_ = |θ_*l*_ – θ_*r*_|. The angles θ_*l*_ and θ_*r*_ are measured by fitting only the left and only the right half of the cylindrical water droplet (Figure 2). In our simulations, Δθ_hist_ does not result from an externally imposed symmetry-breaking field (e.g., gravity on an inclined surface), but rather from unavoidable asymmetries in the initial nanodroplet configuration. Accordingly, while a nonzero Δθ_hist_ is consistent with contact-line pinning, its absence does not exclude pinning.

Figure 6 reports the contact angle hysteresis for the case with a fixed number of hydrophilic spots and increasing hydrophilicity.

**Figure 6:**
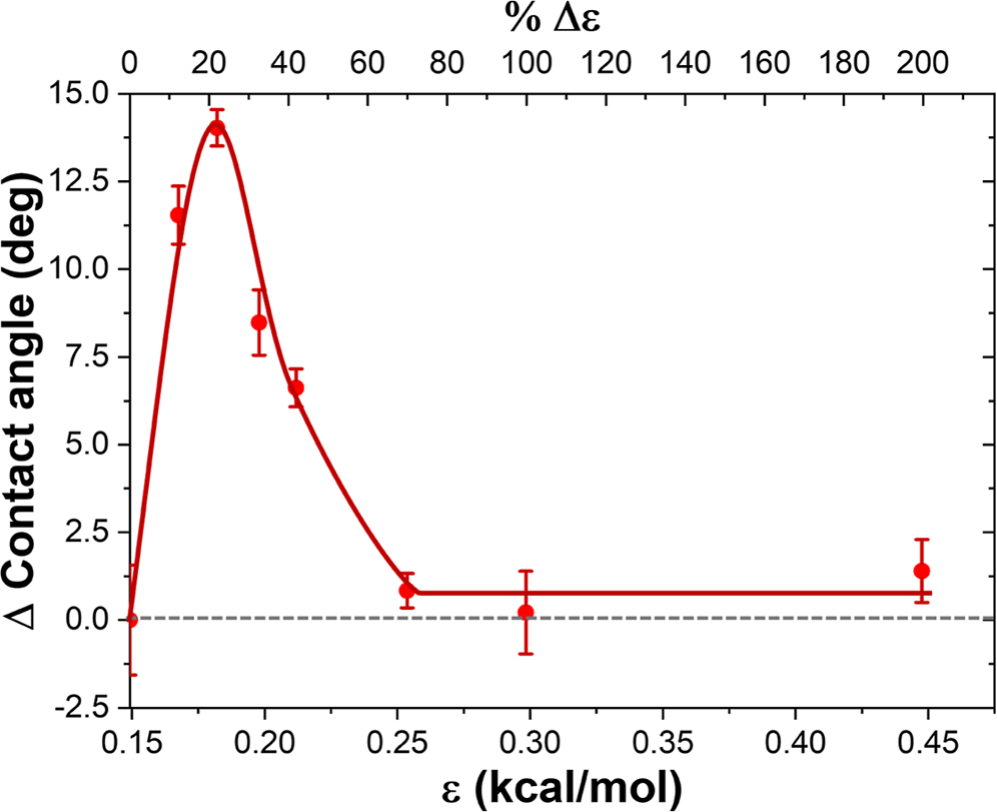
Contact angle hysteresis for the “3-lines” heterogeneous material. The plot shows the difference between the left and right contact angles as a function of the attractive strength ε_LS_^α^. The red line is added to guide the eye and highlights a maximum around Δε_LS_^α^ ≈ 20%. For the absolute value of the contact angle in the two sides of the droplet see Table SI-1.

The contact-angle hysteresis initially increases with the interaction strength between water and the hydrophilic sites, then decreases, reaching a sharp maximum at %Δε ∼ 20%. At %Δε ∼ 70%, it becomes negligible again.

To assess whether pinning is a genuine effect rather than an accidental consequence of the initial droplet position, we performed two sets of validation simulations. The first set of validation simulations were performed according to a deposition protocol consistent with the one adopted with the original droplet: the droplet was thermalized at a distance from the surface with its center of mass shifted by 3 Å to the left and to the right of the original one. A shift of 3 Å corresponds to 30% of the distance between the hydrophilic spots, which are the surface features to which are responsible for the nonmonotonic trend of the contact angle as a function of the spot/water interaction strength. This displacement was chosen to be substantial, while still avoiding a translation that would place the droplet in an initial configuration equivalent to the original one. After thermalization, as in the original protocol, we applied a gentle velocity to deposit the droplet onto the surface. In addition, we performed a second set of simulations in which the droplet, already deposited on the surface in the original configuration, was displaced by 4 Å, corresponding to about 40% of the distance between hydrophilic spots.

In all these cases, we obtained results consistent with the original simulations: both the trends in contact angle and contact-angle hysteresis for all shifted droplets are consistent with those observed for the original droplet (Figures SI-11–SI-14). Importantly, this consistency is not merely qualitative. The contact angle and contact-angle hysteresis measured across the four sets of simulations and the eight values of the hydrophilic–spot interaction strength, for a total of 32 cases, are mutually consistent for corresponding conditions, regardless of the deposition protocol. This demonstrates that our results are not an artifact of an accidental deposition event, but rather reflect a genuine property of the system under investigation.

Despite some quantitative mismatch between the Δθ_hist_ and θ_*Y*_ profiles—for example, Δθ_hist_ reaches its maximum at %Δε ∼ 20%, whereas θ_*Y*_ peaks at %Δε ∼ 40%, and the former maximum is sharper than the latter—the trends in hysteresis and contact angle are clearly correlated, as both quantities exhibit an initial increase followed by a decrease with increasing interaction strength of the hydrophilic sites. This correlation suggests that the unusual behavior of the contact angle arises from the same underlying phenomenon responsible for hysteresis, namely pinning of the contact line. We therefore hypothesize that atomic-scale chemical heterogeneity generates strong three-phase contact-line pinning, which initially increases the contact angle. When the attraction between water and the hydrophilic sites becomes sufficiently strong, however, enhanced wettability overcomes pinning, and the contact angle decreases.

To confirm the triple-line pinning discussed above, we performed simulations to assess the receding contact angle.^66^ In particular, starting from the final configuration of the 3000 water molecule droplet deposited on the substrate used to measure Δθ_hist_, we subtracted 600 water molecules from the top region of the droplet, let the system relax, and measured the contact angle. We focused our attention on three of the cases discussed above: (i) a fully hydrophobic substrate, in which all particles of the substrate have the same interaction with water droplets; (ii) a substrate with +42% enhanced interaction, corresponding to the maximum of the contact angle for a substrate of this kind; and (iii) a substrate with +200% enhanced attractive interaction with water. In the first case, we observe a hysteresis of approximately ∼−2.5°, defined as the difference between the contact angles measured for droplets containing 2400 and 3000 water molecules. Consistently with the Δθ_hist_ trend, for the second case we observe a much larger hysteresis of approximately ∼8.5°. Finally, once again consistently with the Δθ_hist_ trend, for the third case we observe negligible hysteresis. In summary, a direct estimation of the receding contact angle also reveals strong pinning associated with atomic-level chemical heterogeneities.

Pinning is also apparent from visual inspection. Figure 7 shows that the contact line is arrested at moderately hydrophilic sites (Δε_LS_^α^ = +12% and +42%). Notably, a single hydrophilic particle suffices to arrest the contact line at Δε_LS_^α^ = +42%. For more strongly hydrophilic particles, adhesion dominates over pinning, and the contact line is no longer arrested by the hydrophilic spots. Once again, this is not an artifact of an accidental deposition event, as consistent results are also obtained after relaxation of droplets displaced by 4 Å—about 40% of the distance between hydrophilic spots—from the position of the original droplet (Figure SI-15).

**Figure 7:**
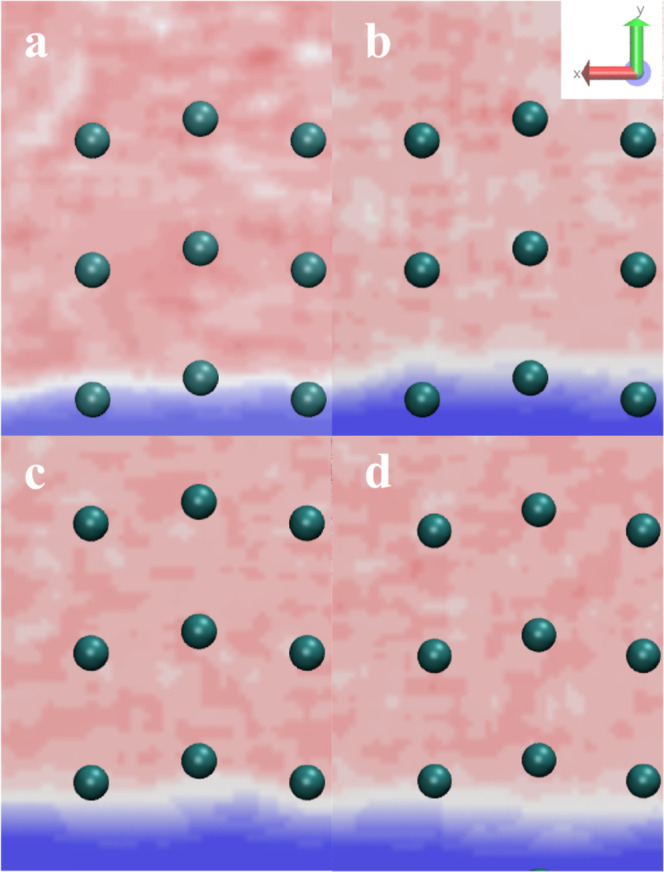
Two-dimensional water density at the solid/liquid interface (*xy* plane). Blue indicates zero water molecule density, red denotes high (bulk) water molecule density. The contact line corresponds to the white stripe. It can be observed that the water droplet is arrested by the hydrophilic particles in the case of Δε_LS_ = +12% (a) and +42% (b). In particular, in the case of Δε_LS_ = +42%, a single hydrophilic particle is sufficient to arrest the contact line. For even strongest hydrophilic particles (Δε_LS_ = +70% (c) and +100% (d)) the contact line is no longer arrested by the hydrophilic spots.

To assess whether the contact angles of the other molecular sizes, shown in Figure SI-10, were also affected by contact-line pinning, we estimated the hysteresis in its approximate form, namely as the difference between the left and right contact angles. As shown in Figure SI-16, a similar trend to that observed in Figure 6 is found also in this case. Although the absolute values differ, in the region where the increase in the contact angle is observed, the corresponding difference between the left and right contact angles remains non-negligible, further supporting the presence of contact-line pinning effects in nanoscopic domains.

### 3.2 Contact Angle vs Number of Hydrophilic Spots

We consider a heterogeneous surface with an increasing number of hydrophilic particles, ranging from 0% to 100%, that is, from a surface composed entirely of hydrophobic particles (θ_*Y*_ ∼ 99°) to one composed entirely of hydrophilic particles (θ_*Y*_ ∼ 85°). For hydrophilic particles we set ε = 0.21186 kcal mol^–1^, i.e., an interaction with water that is ∼42% stronger than that of the hydrophobic particles. Also in this case, contrary to intuition, one notices that the contact angle initially increases with the number of hydrophilic spots, reaching a maximum when ∼20% of the surface is decorated with hydrophilic particles (Figure 8). Beyond this concentration the contact angle decreases, although its value remains essentially constant (within the statistical error) in the range 75–100%.

**Figure 8:**
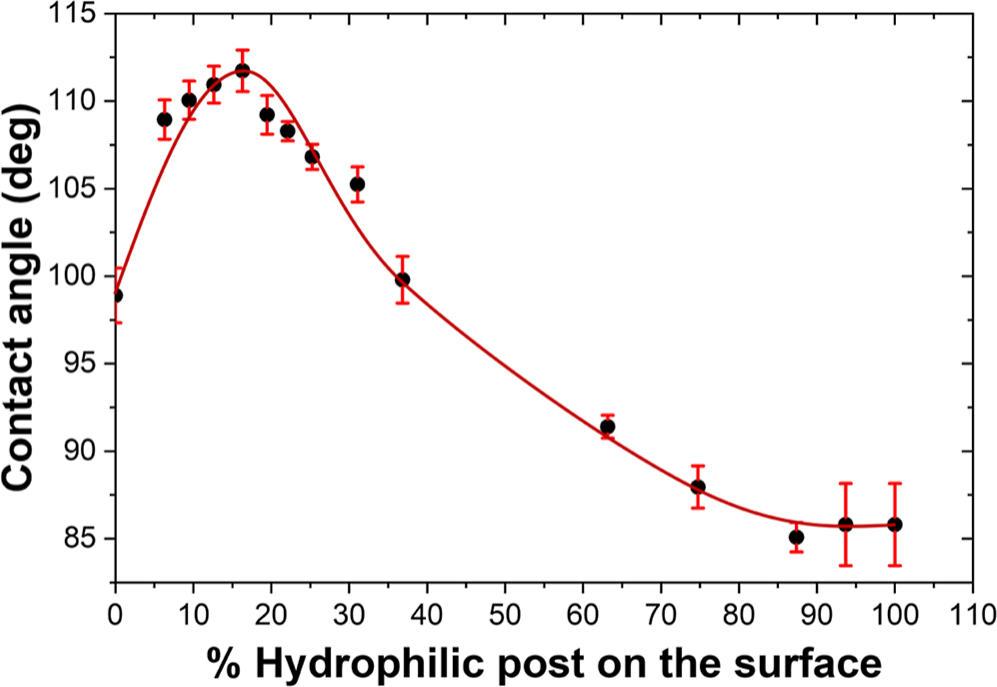
Contact angle vs percentage of hydrophilic particles. Two fixed interaction strengths are employed: ε = 0.14920 kcal mol^–1^ for hydrophobic particles and ε = 0.21186 kcal mol^–1^ for hydrophilic particles. The red line is included only to guide the eye.

To investigate the origin of this trend we follow the approach used in the previous section: (i) we compute the density profile of water as a function of the distance from the surface for various concentrations of hydrophilic particles, and (ii) we compare changes in the characteristic features of these profiles. Again, we observe a trend of the maxima (and minima) of the density profile with the content of hydrophilic/hydrophobic particles rather than a trend correlating with the contact angle, which exhibits a maximum at 20% hydrophilic particles (Figure 9).

**Figure 9:**
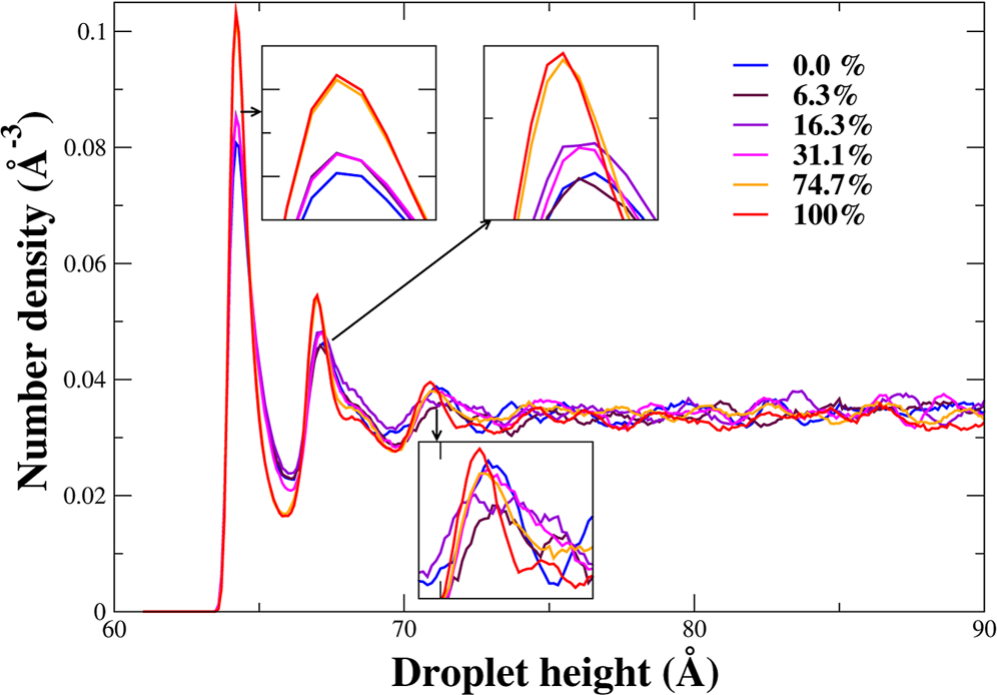
Density field of water oxygens for the same ε_LS_^α^ while varying the number of hydrophilic spots on the surface.

Based on the preceding analysis of contact angle hysteresis, we attribute this behavior to a trade-off between the overall hydrophilicity of the material and triple-line pinning induced by the hydrophilic particles.

### 3.3 Summary and Discussion of Results

The observed dependence of the contact angle on the number and interaction strength of hydrophilic sites shows that a surface can remain hydrophobic even in the presence of atomistically sized hydrophilic patches. In other words, the present results suggest that a moderate amount of hydrophilic functionality can preserve, and even stabilize, the nominal hydrophobicity of a surface.

These findings may open new perspectives for surface and pore design. On the one hand, they suggest new chemical-engineering principles for the design of surfaces and cavity walls that repel liquid water while still allowing interaction with water vapor. On the other hand, they may help rationalize counterintuitive wetting behavior in porous materials. For example, they may explain why Cu_2_(tebpz) remains highly hydrophobic despite the presence of hydrophilic Cu ions in its cavities, while still exhibiting a high water-vapor uptake prior to intrusion.^25^ They may also help explain why the intrusion and extrusion pressures of Cu_2_(tebpz) and ZIF-8 increase with temperature, that is, why wetting of their cavities becomes more difficult even though their nominal hydrophobicity decreases with temperature.^25^ Of course, the present results do not rule out the possibility that the peculiar properties of Cu_2_(tebpz) are also influenced by other structural characteristics of the metal–organic framework, such as the lateral apertures in the pore walls.^67^

More broadly, our findings also inform discussions of the hydrophobic or hydrophilic character of more complex surfaces composed of atomistic or molecular units with different affinities for water, such as proteins. In this context, the character of specific domains is often assessed using hydrophobicity scales defined for individual amino acids.^68,69^ However, consistently with recent literature,^31–33^ our results suggest that the hydrophobicity of individual amino acids is not, by itself, a reliable predictor of the hydrophobicity of a protein domain.

These results further inform the discussion on whether the contact angle is an adequate descriptor of the hydrophobic character of a surface. Indeed, for the nanodroplets considered here, we find that a surface decorated with 20% strongly hydrophilic sites corresponds to the maximum contact angle. Moreover, up to 40% of such hydrophilic sites can be introduced on an otherwise hydrophobic surface before the contact angle decreases to the value measured for the purely hydrophobic case. One may therefore expect that, as in the case of Cu_2_(tebpz),^24^ while liquid water is effectively repelled by the surface, individual water molecules in the vapor phase may still be attracted to the hydrophilic sites. We refrain from drawing firm conclusions on this point from the limited number of cases considered in this work. Nevertheless, we believe that our results provide an important contribution to the broader discussion of contact angle as a descriptor of hydrophobicity.

Before extending the present results to a broader range of solid–liquid interfacial systems, several questions remain to be addressed. First, the dependence of the observed phenomenology on the patterning of the hydrophilic sites requires further investigation. In this work, we considered a family of patterns defined by the number of rows of hydrophilic sites, all derived from the same topology inspired by the arrangement of copper atoms in Cu_2_(tebpz). A systematic study of both regular and random patterning topologies will therefore be necessary in future work. In addition, given the observation of Ozcelik et al.^59^ that pinning is governed by the ratio between droplet size and the characteristic length scale of the surface patterning, the geometrical features of the surface—for example, the distance between hydrophilic sites within a given topology—also need to be investigated.

Another important question concerns the droplets’ size dependence of the observed phenomenology. In this work, we have shown that, because of pinning at hydrophilic sites, the absolute value of the contact angle depends on droplet size. By extension, one may expect the absolute value of the contact angle to depend on the size of the liquid front in confined systems, which is set by the cavity size. Moreover, although we have shown that the qualitative features discussed here persist for droplets of different sizes, it remains to be established whether the same conclusion holds for other hydrophilic-site patterning topologies.

A systematic investigation of the broad range of possible combinations of surface topology, surface geometry, and droplet size may prove prohibitively expensive by molecular dynamics. A mesoscale analysis that accounts for the combined effects of adhesion strength and three-phase contact-line pinning on the overall hydrophobic or hydrophilic character of a macromolecular surface may therefore offer a useful compromise between accuracy and computational efficiency, enabling a more comprehensive exploration of the relevant characteristics of the solid–liquid system. In practice, one could resort to classical density functional theory or Cahn–Hilliard-type modeling of the interfacial system. However, before these approaches can be applied with confidence, their ability to capture the phenomenology discussed in this work and to represent fluid–surface interactions reliably must first be assessed.

## 4 Conclusions

We have investigated the contact angle of surfaces with atomistic-level heterogeneities—systems composed of atoms and molecules with differing affinities for water, such as the internal walls of certain metal–organic frameworks. Rather than focusing on a specific material, we examined a simpler model system in which particles interact with water molecules via a Lennard-Jones potential. In this work, the surface pattern topology and geometry of hydrophilic spots is consistent with that of copper atoms in Cu_2_(tebpz). Two scenarios were considered: (i) a *hydrophobic* surface decorated with a fixed number of regularly distributed *hydrophilic* particles whose interaction strength with water is varied; and (ii) an *otherwise hydrophobic* surface containing a variable fraction of *hydrophilic* particles of fixed interaction strength.

Contrary to intuition, decorating a hydrophobic surface with hydrophilic spots does not necessarily make the surface less hydrophobic (or more hydrophilic). For example, when the number of hydrophilic particles is varied, maximum hydrophobicity is observed at a concentration of ∼20% hydrophilic particles. This effect cannot be explained by a simple, monotonic modulation of hydrophobicity by the hydrophilic particles: for chemically homogeneous surfaces, the contact angle shows a monotonic dependence on the water–particle interaction strength.

On the one hand, this result provide a possible explanation of why highly hydrophobic surfaces, such as the inner walls of Cu_2_(tebpz), can still contain hydrophilic sites (Cu^+^ ions in that example). On the other hand, it raises new questions about how we interpret and design the properties of biological and nonbiological surfaces, which typically exhibit atomic or molecular heterogeneities. Notably, our findings show that homogenization arguments cannot be applied at this scale, challenging the use of simplified continuum models to represent atomistic or molecular systems.

To rationalize the unusual trends, we hypothesized that the counterintuitive maximum in contact angle for chemically heterogeneous surfaces arises from two competing effects of the hydrophilic spots: (i) increased surface–water adhesion, and (ii) pinning of the three phase contact line. We tested this idea by measuring the hysteresis between the left and right contact angles of cylindrical droplets; a hysteresis value exceeding the measurement error indicates pinning. The hysteresis ranges from 2° to 15° and, strikingly, exhibits a maximum at 20% hydrophilic particles, supporting our hypothesis. This analysis is confirmed by the trend of the receding angle measured in selected cases.

It must be stressed that this phenomenon is likely magnified by the small size of the nanodroplets employed in our simulations: the contribution of triple-line tension, which scales linearly with droplet radius, is larger than for macroscopic droplets, where surface terms scale with the square of the radius. Nevertheless, the effect should be taken into account whenever hydrophobicity or hydrophilicity is assessed at the nanoscale.

Finally, the present results, namely, the nonmonotonic dependence of the contact angle on the strength and number of hydrophilic sites, further inform the discussion on whether contact angle is an adequate descriptor of the hydrophobic character of a surface.

## Supplementary Material





## Data Availability

Input and data files for selected simulations spanning the vast simulation campaign at the basis of this article are available in the Supporting Information. Additional data that support the findings of this study are available from the corresponding author, Andrea Le Donne (andrea.ledonne@unife.it) upon request. Access will be granted to qualified researchers for the purpose of replicating or verifying the results reported in this article.

## Notes

(i) The error-propagation method; (ii) top views of the patterned surfaces and their geometric characteristics; (iii) contact-angle trends and density profiles for the cases with 2 and 4 lines; (iv) contact-angle trends for different nominal positions of the solid surface; (v) contact-angle trends for different droplet sizes (3 lines geometry); (vi) contact angle trend and hysteresis trends for different initial conditions; (vii) contact-angle trend and hysteresis trend for lateral displacement (3 lines geometry); (viii) hysteresis trends for different droplet sizes (3 lines geometry); (ix) the absolute contact-angle hysteresis for the case with 3 lines discussed in the main text; and (x) snapshots of the simulation setup, including the initial droplet deposition, the homogeneous hydrophobic and hydrophilic systems, and the cases with 2 lines at Δε_LS_^α^ = +12% and Δε_LS_^α^ = +200% (PDF)
Droplet deposition (ZIP)

## †

Presently at Institute of Physics and Materials Science, Faculty of Natural Sciences and Technology, Riga Technical University, Paula Valdena 3/7, Riga LT-1048, Latvia
