# Supplementary material for: When 20% Is Enough: Counterintuitive Contact Angle Maxima on Chemically Heterogeneous Hydrophobic/Hydrophilic Surfaces
Source: Langmuir. 2026 May 28;42(23):16191–202. doi: 10.1021/acs.langmuir.6c00428 (PMC13276908; doi:10.1021/acs.langmuir.6c00428)
Supplement: Supplementary file 1 [file la6c00428_si_001.pdf]

SUPPORTING INFO

When 20 % Is Enough: Counter-Intuitive contact angle Maxima on Chemically Heterogeneous Hydrophobic/Hydrophilic Surfaces

Lorenzo Brugnati[1], Andrea Le Donne*[a1], and Simone Meloni[1]

[1]Department of Chemical, Pharmaceutical and Agricultural Sciences, DOCPAS, Universitá di Ferrara, Via Luigi Borsari 46, 44121 Ferrara, Italy.

*Electronic mail: andrea.ledonne@unife.it

Contents

1 **Error estimation** **S3**

2 **Surfaces** **S4**

3 **Contact angle for the "two-line" case** **S7**

4 **Contact angle for the "four-line" case** **S9**

5 **Contact angle for different nominal positions of the surface** **S11**

6 **Contact angle for different droplet sizes** **S13**

7 **Contact angle and contact-angle hysteresis for the "three-line" case, obtained for different initial droplet positions.** **S14**

8 **2D water density at the solid/liquid interface after lateral displacement 3 lines** **S19**

9 **Hysteresis trend 3 lines different droplet size** **S20**

10 **Absolute value of contact angles in the case of 3 lines hydrophilic spots** **S21**

[a]Presently at Institute of Physics and Materials Science, Faculty of Natural Sciences and Technology, Riga Technical University, Paula Valdena 3/7, Riga LT-1048, Latvia

- Number of pages:21
- Number of figures:16
- Number of tables: 1
- Number of schemes: 0

1 Error estimation

To change the hydrophobicity of the material, the value of $\varepsilon_{LS}^{\alpha}$ was varied: the lower the value of $\varepsilon_{LS}^{\alpha}$, the higher the hydrophobicity of the atom. For each hydrophobicity value, the contact angle value was studied using the drop fitting technique. Each simulation lasted 3.0 ns, of which the first 0.5 ns was discarded. This process eliminates the initial frames, which are the noisiest and can lead to errors in determining the contact angle. To estimate the error in the measurement, the trajectory was divided into 15 sub-trajectories, whose frames were combined first by a systematic pattern, according to the value of the unit digit (e.g. 1-16-31-...; 0-15-30-...) and then by random sampling. In all the sub-trajectories the contact angle was determined manually by fitting. Then, the error was determined according to the leave-one-out jackknife procedure over the 15 sub-trajectories, which accounts for temporal correlations intrinsic to molecular dynamics simulations. As an independent check, bootstrap resampling over the same 15 sub-trajectories was also performed; bootstrap provides an empirical sampling distribution for the mean and a complementary confidence interval estimate. Where jackknife and bootstrap gave similar values we report the jackknife result as the main uncertainty; where they differed, the more conservative estimate was reported. The number of sub-trajectories was limited to 15 due to the significant manual effort required by the droplet fitting procedure. Each fit involves human supervision and parameter tuning, making a substantially larger number of independent fits unpractical within reasonable time constraints.

2 Surfaces

The following images are the material used in the simulation. The view is from the plane xy, and the value of sides are 34 for that on the x-axis and 126 for the one on the y. In order we have: $2, 3, 4, 5, 7, 8, 10, 12$ lines.

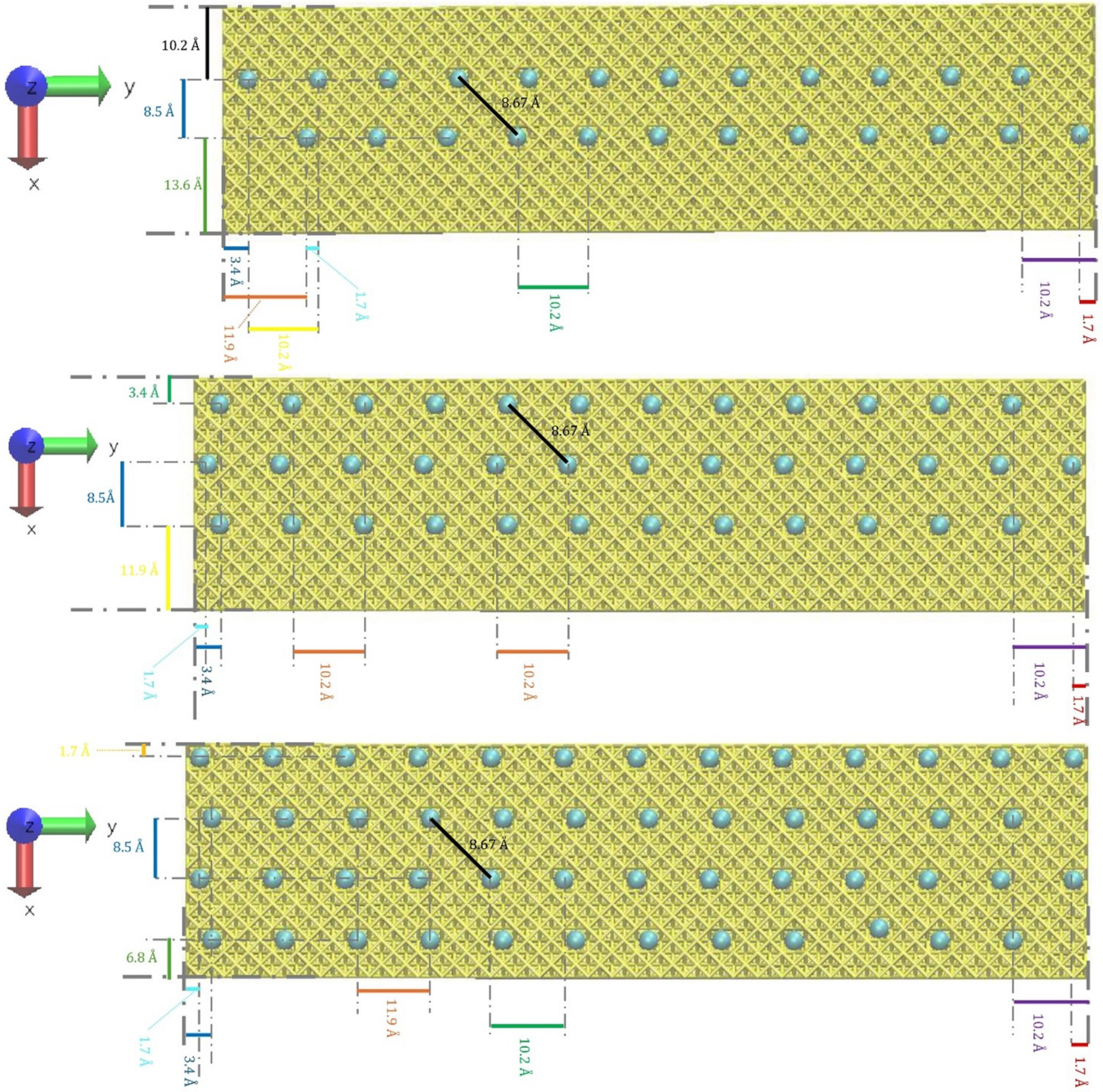

Figure SI-1: Scheme of hydrophobic material with different hydrophilic spot lines number on the surface of the material. In order from first to last there are: 2 lines, 3 lines and 4 lines. These cases are more similar to the real case of Cu_2(tebpz),

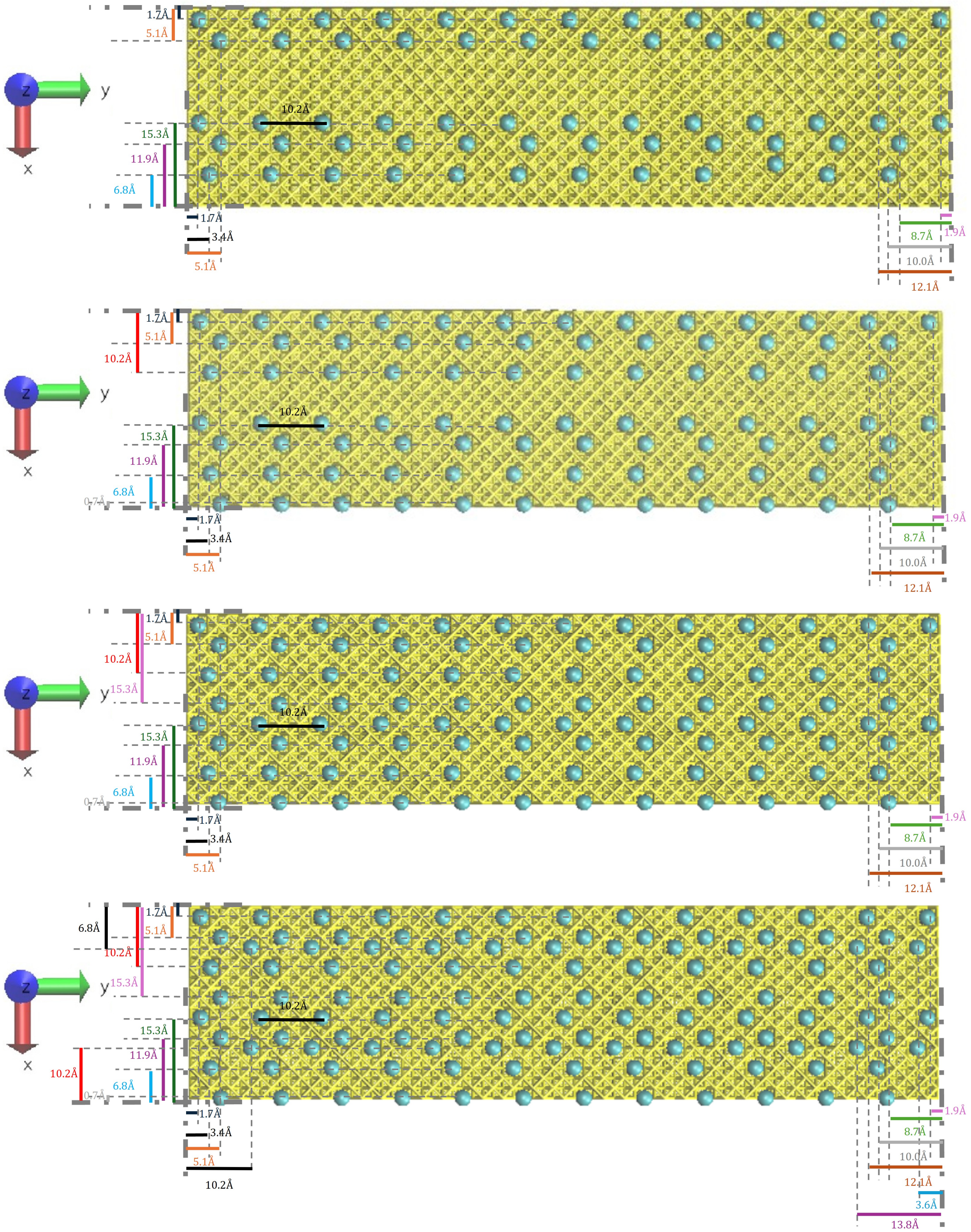

Figure SI-2: Scheme of hydrophobic material with different hydrophilic spot lines number on the surface of the material. In order from first to last there are: 5 lines, 7 lines, 8 lines and 10 lines.

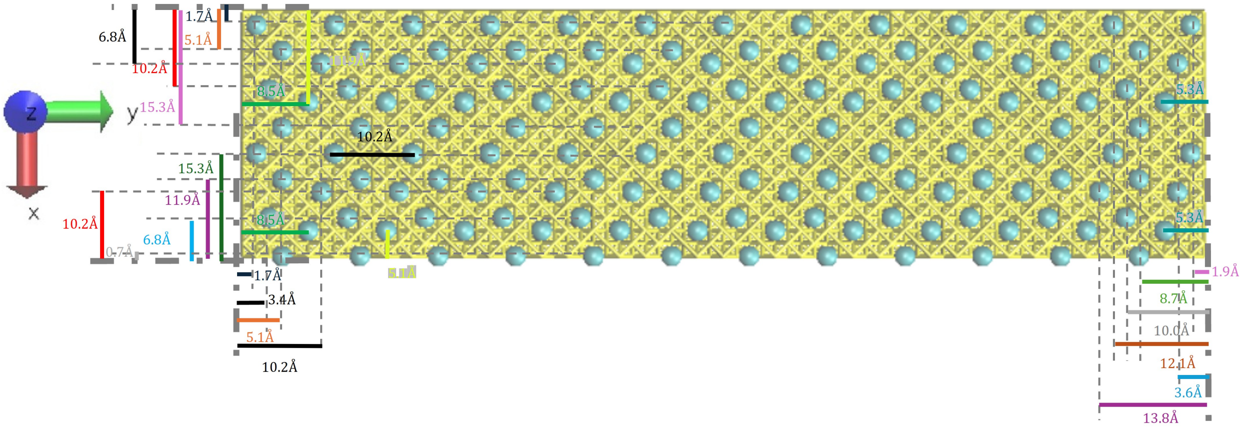

Figure SI-3: Scheme of hydrophobic material with 12 lines of hydrophilic spot on the surface of the material.

3 Contact angle for the "two-line" case

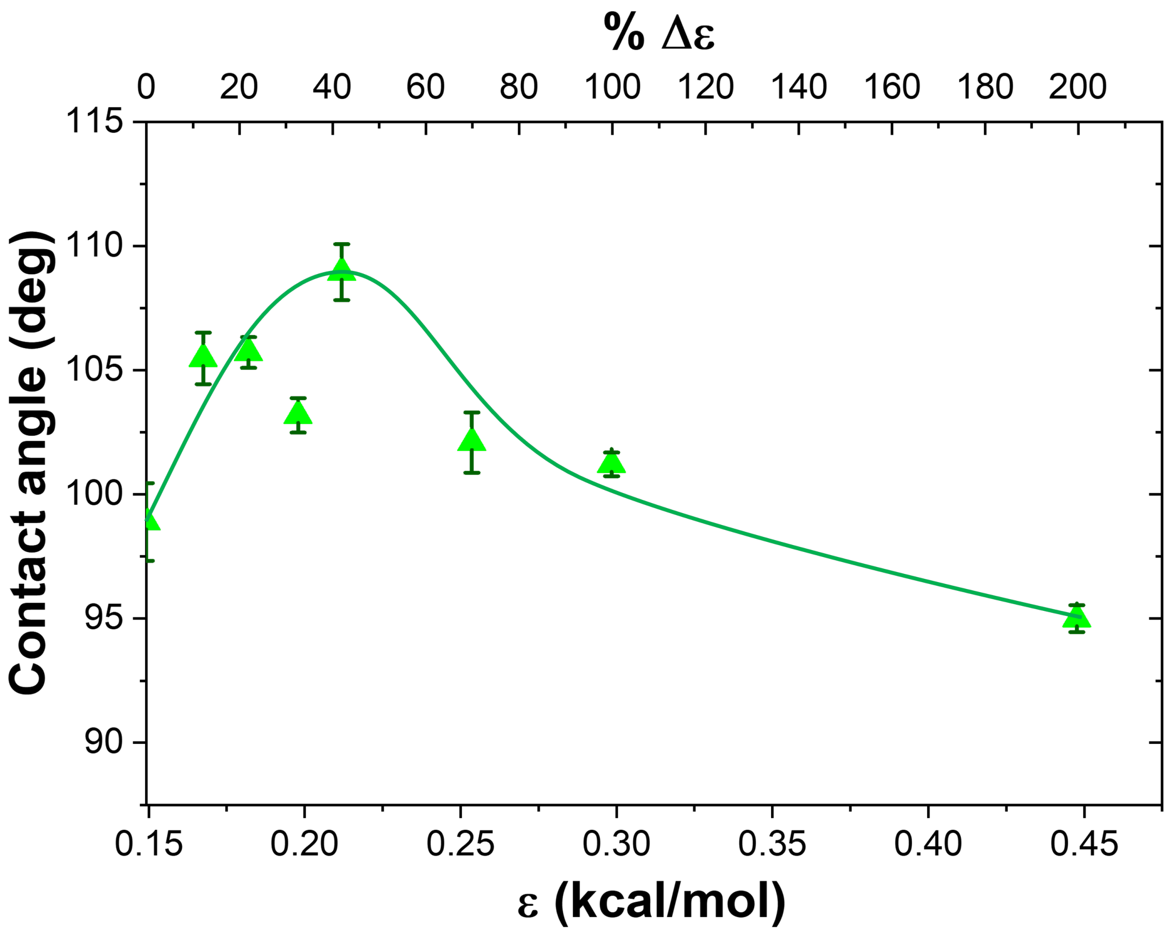

Figure SI-4: Contact-angle trend for the two-line spot configuration as the hydrophilicity of the spots increases. The percentage variations are given relative to the initial value $\varepsilon_{LS}^{\alpha} = 0.14920\,\text{kcal/mol}$. The green line is included only as a visual guide to the trend. An overall increase in the contact angle is observed over the range $\Delta\varepsilon_{LS}^{\alpha} \in [0\%, 50\%]$, although the trend is non-monotonic.

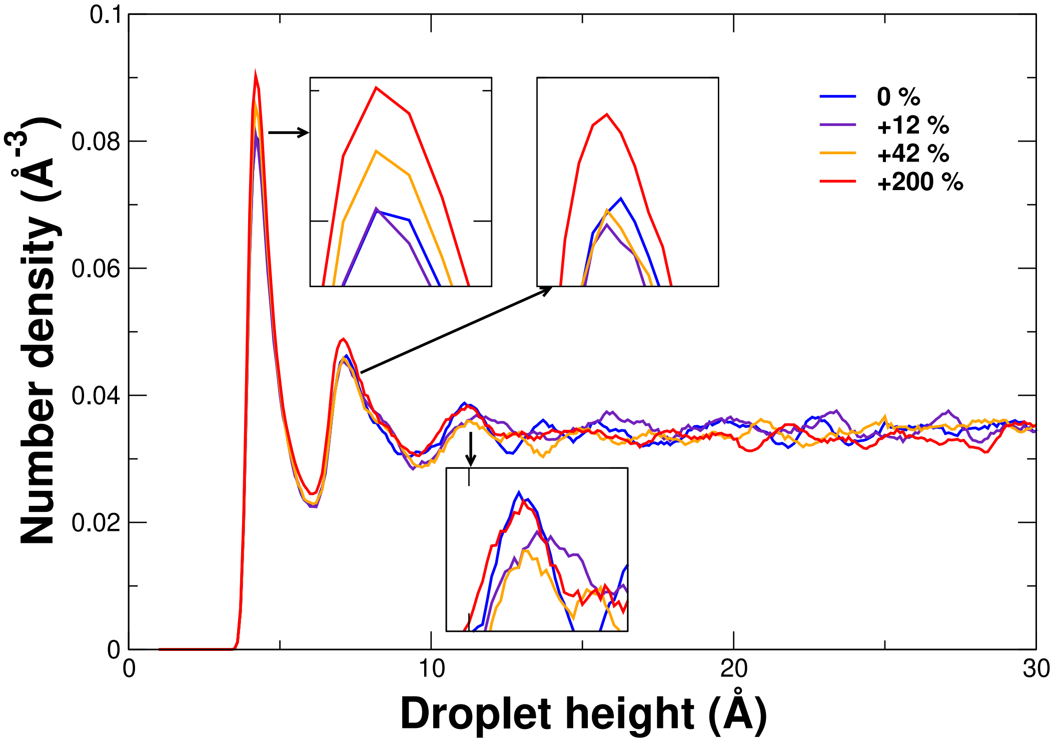

Figure SI-5: Density field of water oxygen atoms in the two-line spot configuration as a function of the value of $\varepsilon_{LS}^{\alpha}$. The typical layered structure of a liquid in contact with a solid is observed. While the contact angle shows a non-monotonic behavior, the density field displays the expected monotonic trend. Therefore, no direct correlation is found between the regular evolution of the density profile and the unexpected trend in the contact angle. As in the three-line case, this behavior reflects a competition between triple-line pinning and adhesion forces.

4 Contact angle for the "four-line" case

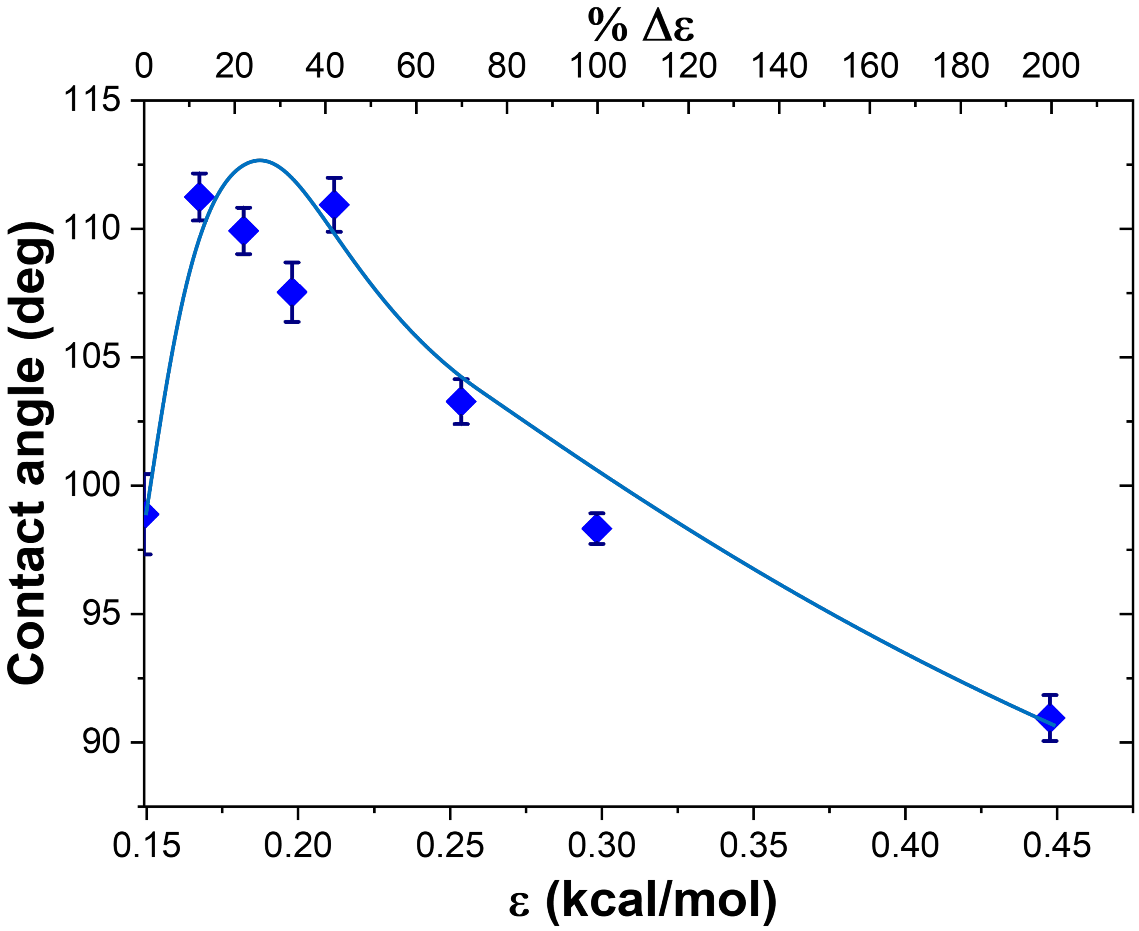

Figure SI-6: Contact-angle trend for the four-line spot configuration as the hydrophilicity of the spots increases. The percentage variations are given relative to the initial value $\varepsilon_{LS}^{\alpha} = 0.14920\,\text{kcal/mol}$. The blue line is included only as a visual guide to the trend. A rapid increase in the contact angle is observed over the range $\Delta\varepsilon_{LS}^{\alpha} \in [0\%, 15\%]$, followed by a decrease over the range $\Delta\varepsilon_{LS}^{\alpha} \in [15\%, 30\%]$. At $\Delta\varepsilon_{LS}^{\alpha} = 42\%$, the contact angle reaches a value close to that observed at $\Delta\varepsilon_{LS}^{\alpha} = 12\%$. Overall, the trend is non-monotonic.

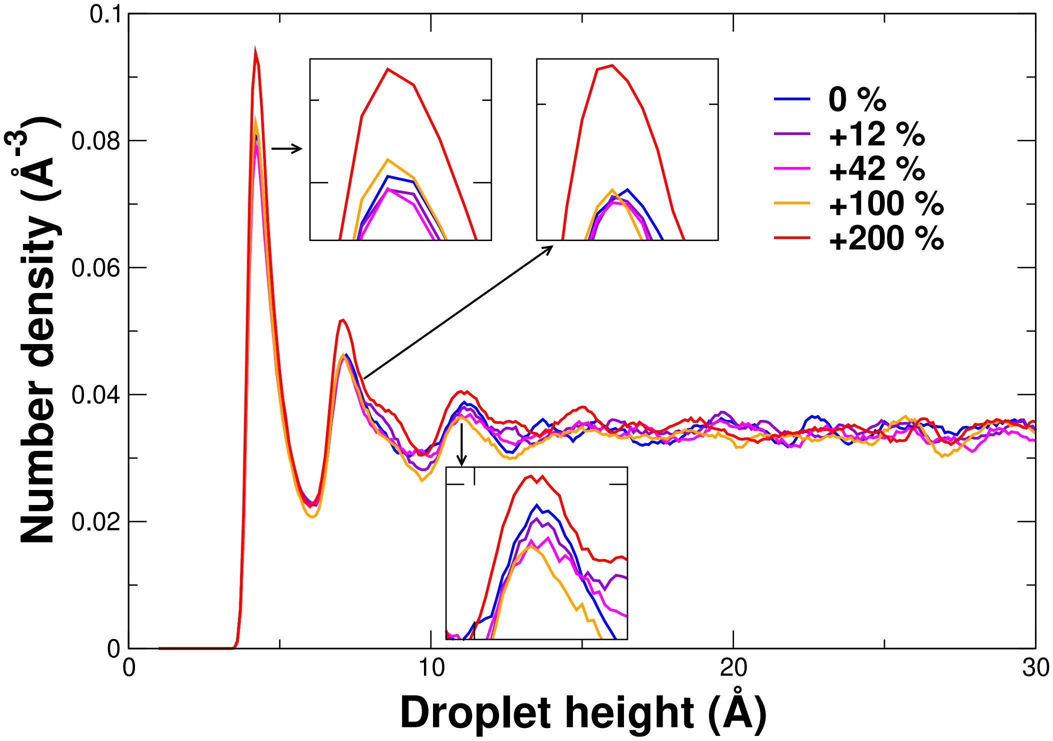

Figure SI-7: Density field of water oxygen atoms in the four-line spot configuration as a function of the value of $\varepsilon_{LS}^{\alpha}$. Also in this case, the characteristic layered structure is clearly observed, and the overall trend is analogous to that found in the other configurations. Although a slight non-monotonic behavior may appear, it can reasonably be attributed to noise. As in the previous cases, the trend in the contact angle as a function of $\varepsilon_{LS}^{\alpha}$ reflects a balance between triple-line pinning and adhesion forces.

5 Contact angle for different nominal positions of the surface

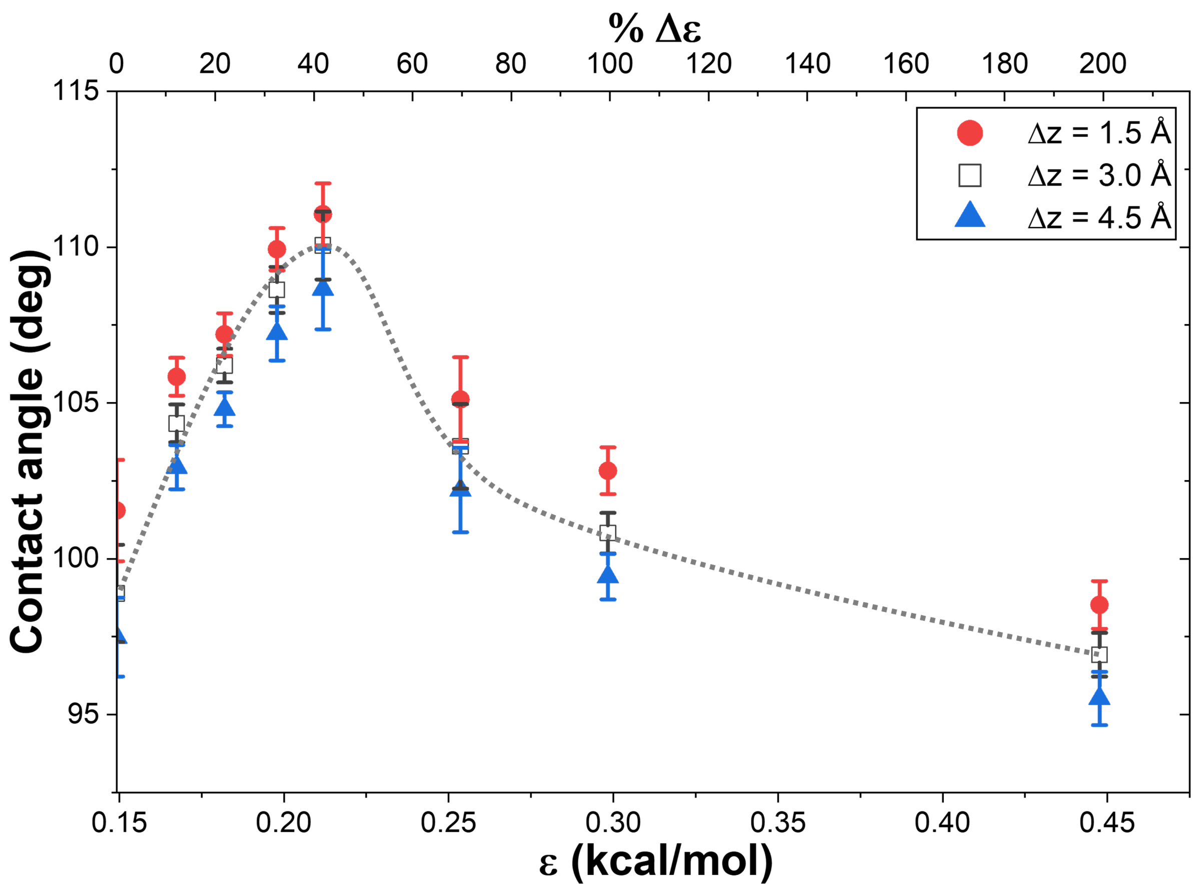

Figure SI-8: Contact-angle trend for the three-line surface as a function of the strength of the hydrophilic spots. Percentage variations are given relative to the reference value $\varepsilon^{\alpha}_{LS} = 0.1490$ kcal/mol. Open squares, together with the dotted gray guideline, correspond to the trend obtained when the nominal solid surface is placed at 3.0 Å from the outermost solid layer, as in the main text. Red circles and blue triangles correspond to nominal solid-surface positions of 1.5 and 4.5 Å, respectively. The figure shows that varying the position of the nominal solid surface within a reasonable range slightly changes the absolute values of the contact angle, but leaves the overall trend unchanged.

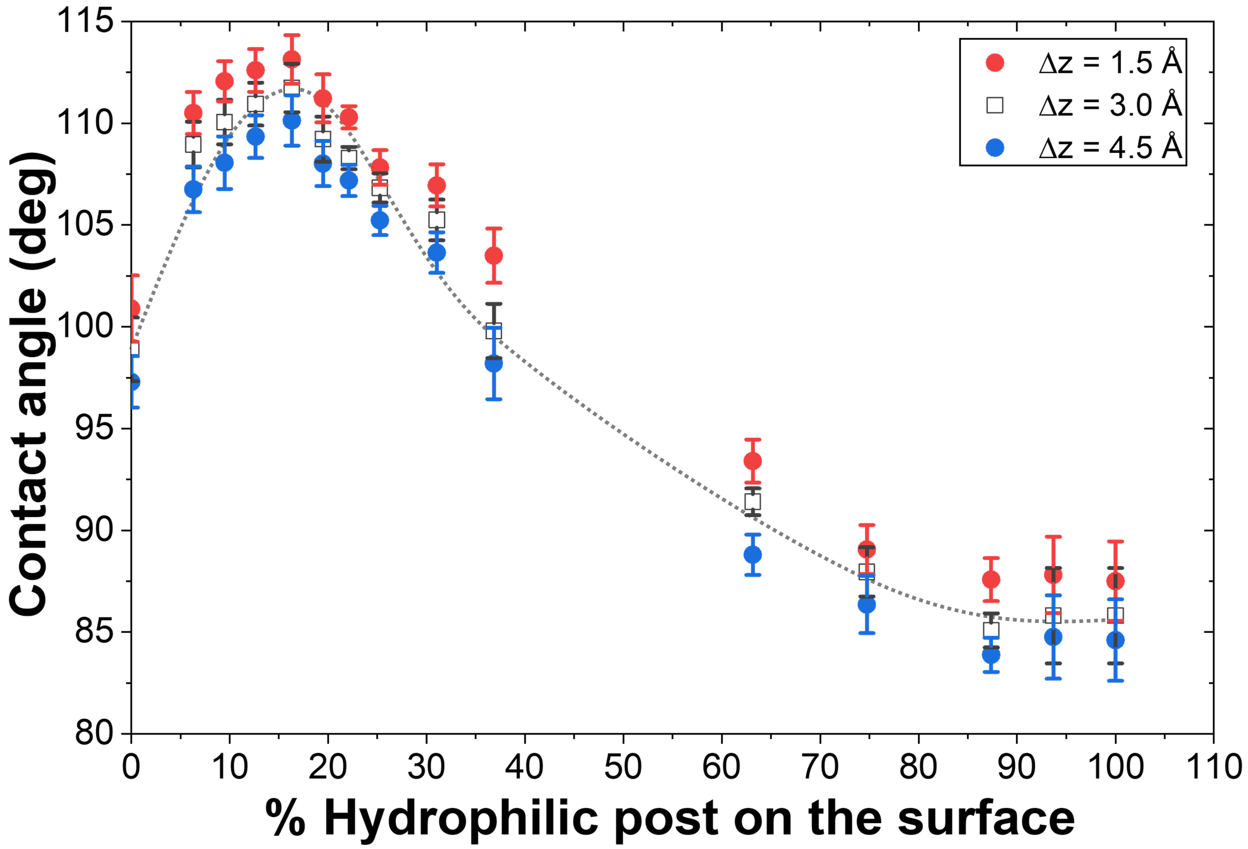

Figure SI-9: Contact angle as a function of the number of hydrophilic spots at $\varepsilon_{LS}^{\alpha}$ = 0.21186 kcal/mol. Open squares, together with the dotted gray guideline, correspond to the trend obtained when the nominal solid surface is placed at 3.0 Å from the outermost solid layer, as in the main text. Red circles and blue triangles correspond to nominal solid-surface positions of 1.5 and 4.5 Å, respectively. The figure shows that varying the position of the nominal solid surface within a reasonable range slightly changes the absolute contact-angle values, but leaves the overall trend unchanged.

6 Contact angle for different droplet sizes

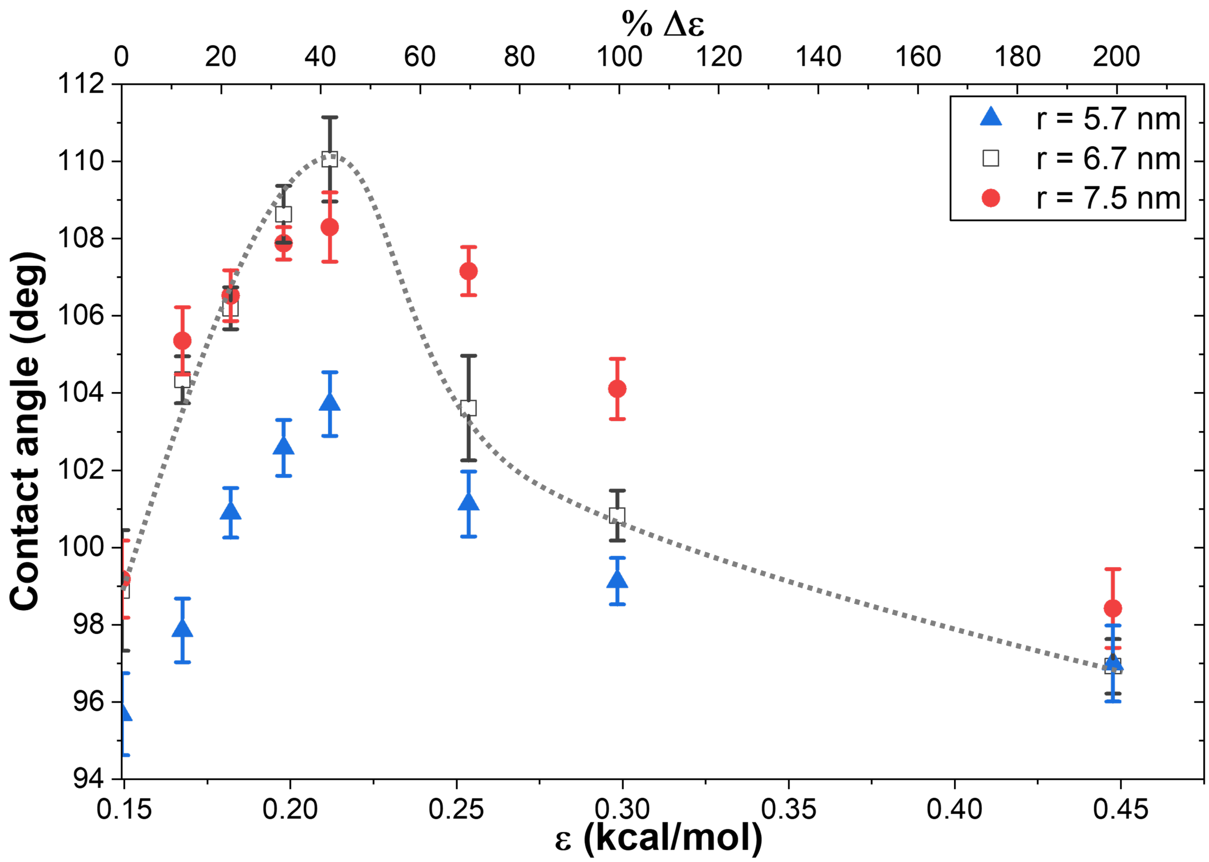

Figure SI-10: Contact-angle trend as a function of the strength of the hydrophilic spots. Percentage variations on the top axis are given relative to the reference value $\varepsilon^{\alpha}_{LS} = 0.1490$ kcal/mol. Open squares, together with the dotted gray guideline, correspond to the droplet size used in the main text. Red circles and blue triangles denote results obtained for droplets that are 50 % larger and 20 % smaller, respectively. As noted in the main text, in the absence of pinning a cylindrical droplet would show negligible size dependence of the contact angle. Here, instead, the absolute contact-angle values depend appreciably on droplet size. Nevertheless, the non-monotonic trend with interaction strength is preserved, and in all cases the maximum remains located at $\Delta\varepsilon = +42$ %..

7 Contact angle and contact-angle hysteresis for the "three-line" case, obtained for different initial droplet positions.

A total of four sets of simulations were performed, each considering eight different values for the interaction strength between the hydrophilic spots and water; these correspond to the values discussed in the main text for the three-line configuration. The first three sets utilized the "dripping protocol": one corresspond to resuls reported in the main text, while the other two are shifted by 3 Å (corresponding to 30% of the distance between the hydrophilic spots) to the left or right relative to the deposition point reported in the main text, after relaxation of positions and velocities initially extracted randomly (Figs. SI-11, SI-12). The fourth set involved an asymmetrical displacement of the original droplet, already deposited on the surface, along the y direction. Specifically, we displaced the droplet by 4 Å (corresponding to 40% of the distance between the hydrophilic spots), such that the translation would avoid obtaining a configuration equivalent to the one in the main text (Figs. SI-13, SI-14). Despite the initial differences in position, after a short relaxation the triple line still exhibits pinning at the hydrophilic spots (Fig. SI-15), just as in the original simulations before the substantial displacement was applied (Fig. 7). All four sets show consistent trends in both contact angle and hysteresis, confirming that pinning at the hydrophilic spots is a genuine phenomenon and not an accidental consequence of the initial placement of the droplet.

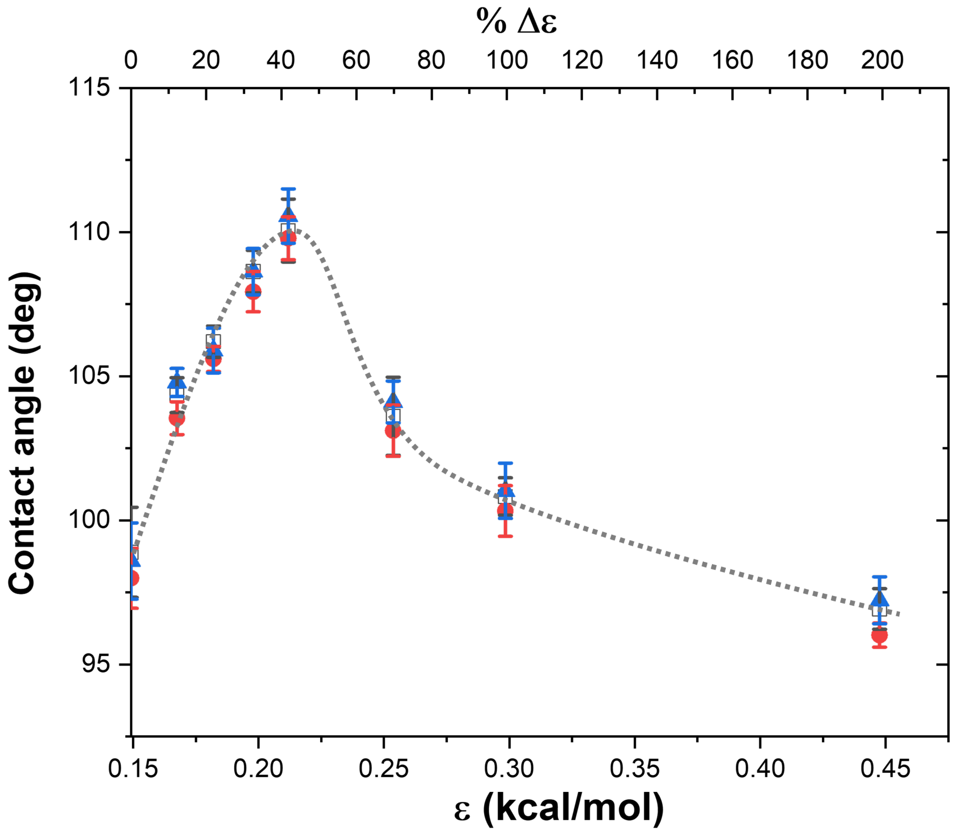

Figure SI-11: Contact-angle trend as a function of the strength of the hydrophilic spots for the three-line case. Percentage variations on the top axis are given relative to the reference value $\varepsilon_{LS}^{\alpha} = 0.1490$ kcal/mol. Open squares, together with the dotted gray guideline, correspond to the droplet initial position used in the main text. Red circles and blue triangles denote results obtained from independent simulations performed with slightly different initial conditions, namely displacements of the droplet center of mass prior to deposition 3 Å on the left and on the right of the original droplet, together with different initial particles' configurations and velocities. The consistency of the results shown in the image confirms that the results discussed in the main text are not artifacts of the simulation protocol.

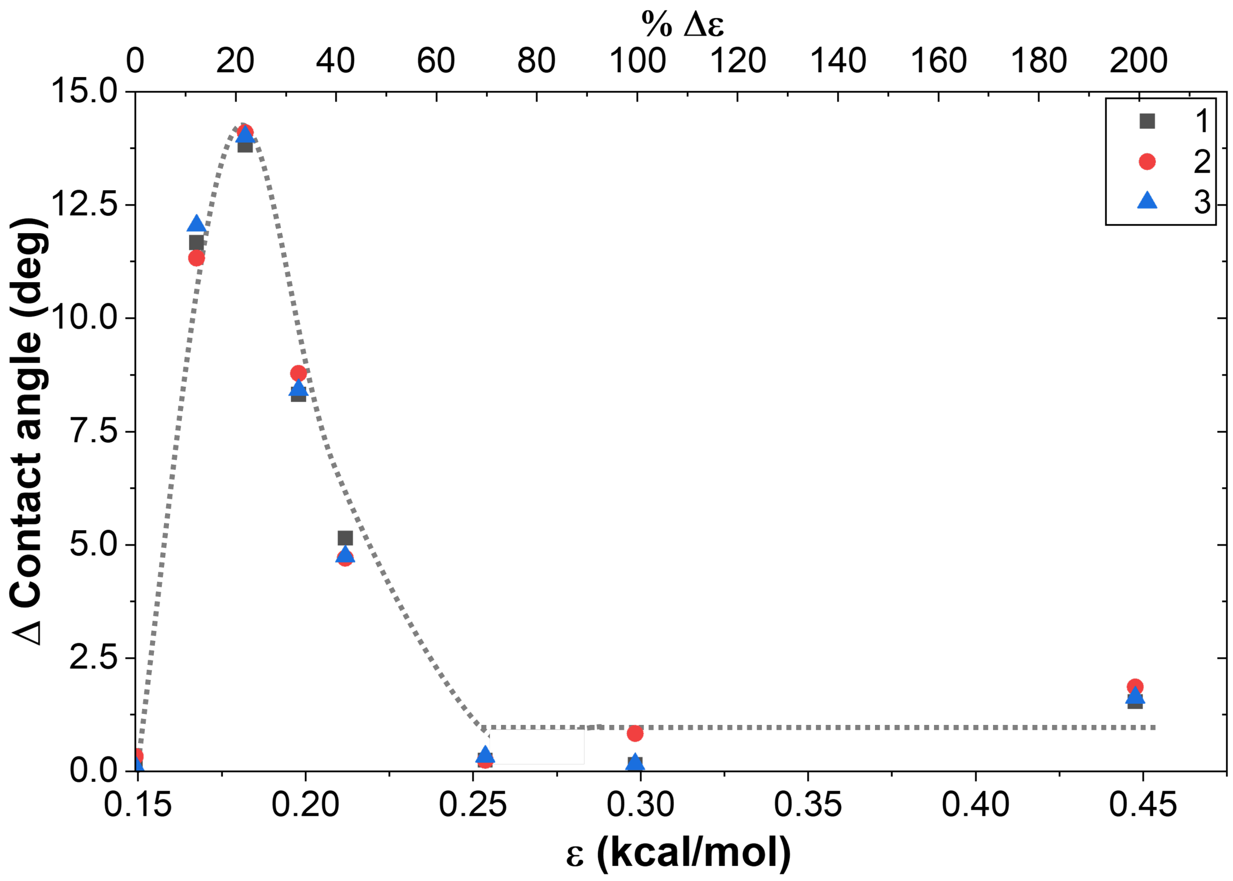

Figure SI-12: Contact-angle hysteresis for the three-line surface. The plot shows the absolute value of the difference between the left and right contact angles as a function of the attractive strength $\varepsilon_{LS}^{\alpha}$. Percentage variations on the top axis are given relative to the reference value $\varepsilon_{LS}^{\alpha} =$ 0.1490 kcal/mol. The dotted light-gray line reproduces the guideline shown in the corresponding figure in the main text (black open squares). Red circles and blue triangles denote hysteresis values obtained from independent simulations performed with slightly different initial conditions, namely 3 Å displacements of the droplet center of mass on the left and right of the oirginal droplet prior to deposition, together with different initial configurations and velocites. This confirms that the results discussed in the main text are not artifacts of the simulation protocol.

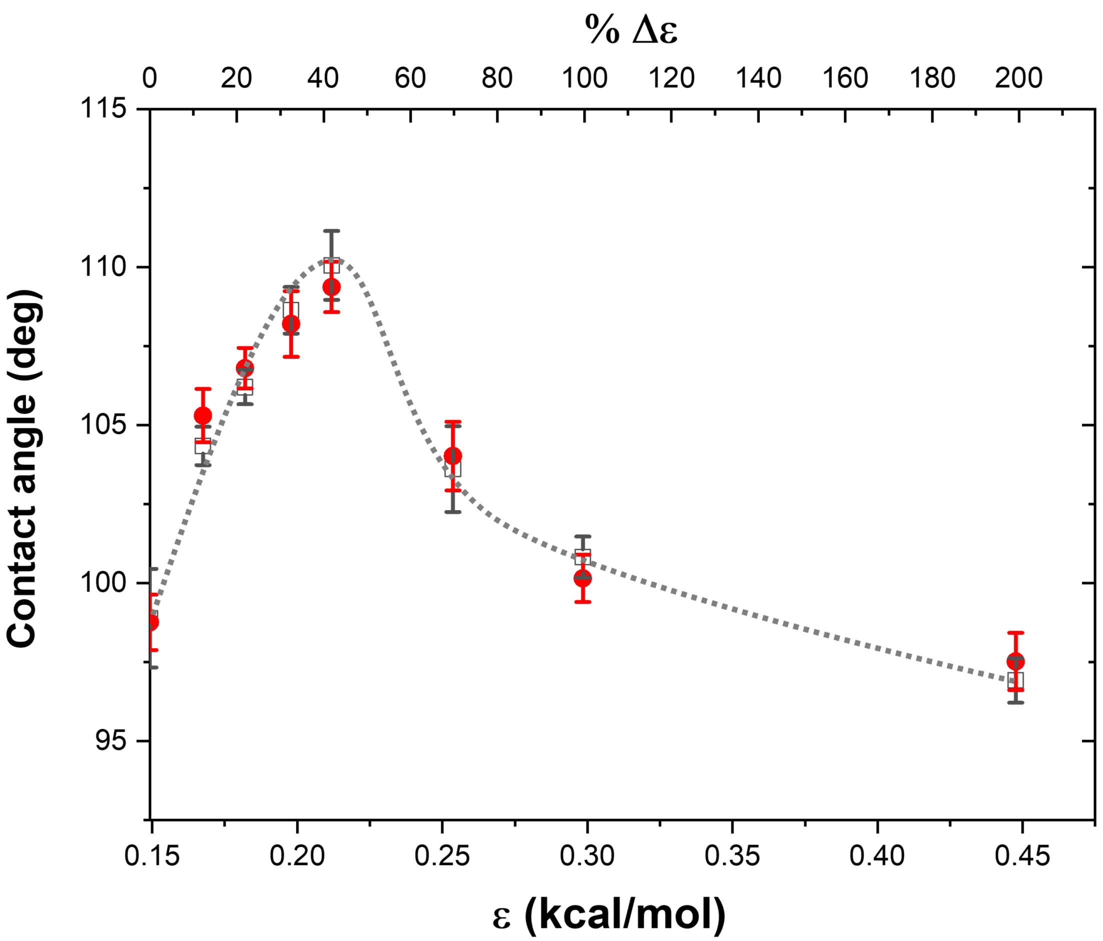

Figure SI-13: Contact-angle trend as a function of the strength of the hydrophilic spots for the three-line case. Percentage variations on the top axis are given relative to the reference value $\varepsilon_{LS}^{\alpha} = 0.1490$ kcal/mol. Open squares, together with the dotted gray guideline, correspond to the droplet configuration used in the main text. Red circles denote results obtained from simulations in which a 4 Å displacement was applied to the original water droplet, moving the triple line into an asymmetric position between hydrophilic spots.

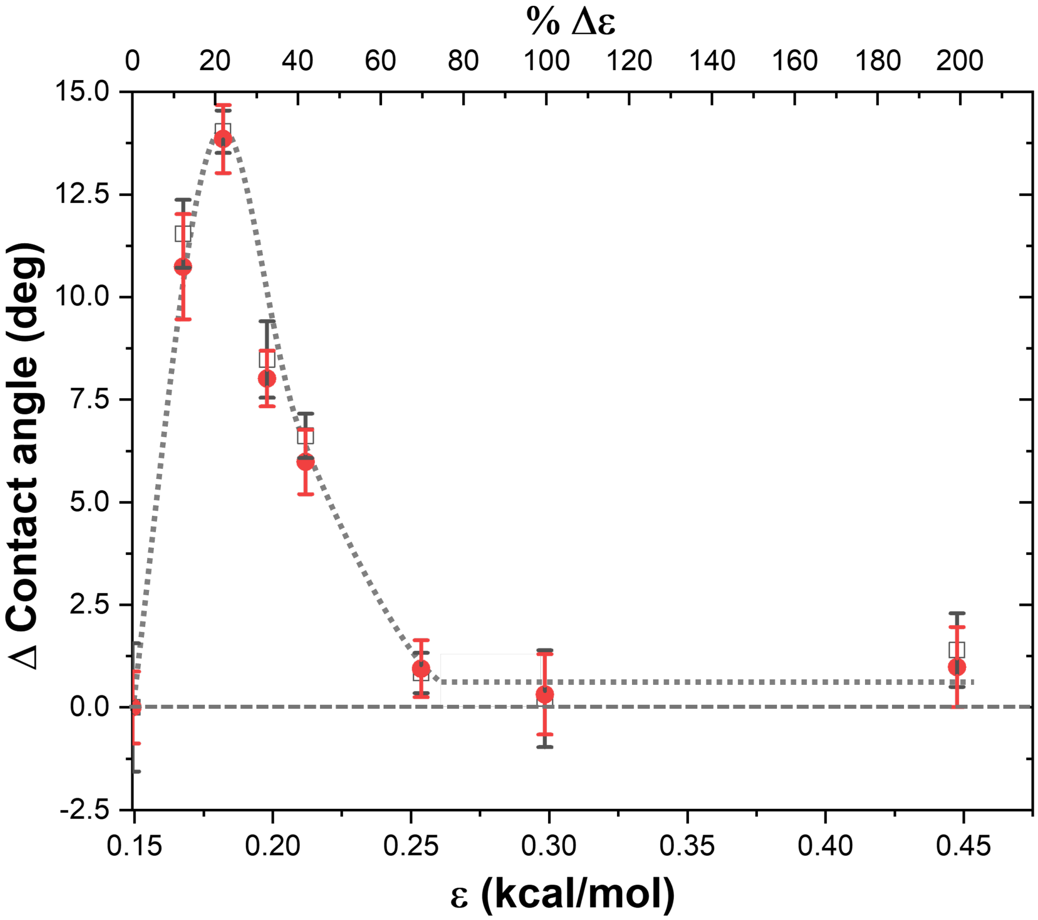

Figure SI-14: Contact-angle hysteresis for the three-line surface. The plot shows the absolute value of the difference between the left and right contact angles as a function of the attractive strength $\varepsilon_{LS}^{\alpha}$. Percentage variations on the top axis are given relative to the reference value $\varepsilon_{LS}^{\alpha} = 0.1490$ kcal/mol. The dotted light-gray line reproduces the guideline shown in the corresponding figure in the main text (black open squares). Red circles denote hysteresis values obtained from simulations in which a 4 Å displacement was applied to the original water droplet, moving the triple line into an asymmetric position between hydrophilic spots.

8 2D water density at the solid/liquid interface after lateral displacement 3 lines

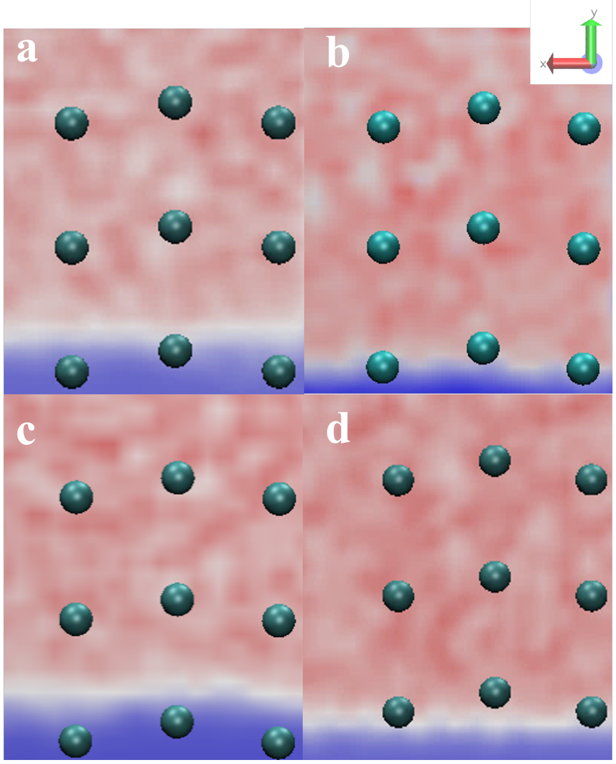

Figure SI-15: Two-dimensional water density map at the solid/liquid interface (the xy plane) for the three-line case after lateral 4 Ådisplacement of the original droplet. Blue regions indicate zero water density, whereas red regions correspond to the bulk water density. The contact line is represented by the white stripe. The figure shows that the water droplet remains pinned at the hydrophilic particles for $\Delta\varepsilon_{LS} = +12\%$ (a) and $\Delta\varepsilon_{LS} = +42\%$ (b). In particular, at $\Delta\varepsilon_{LS} = +12\%$, a single hydrophilic particle is sufficient to arrest the contact line, whereas at $\Delta\varepsilon_{LS} = +42\%$, all three hydrophilic particles participate in the pinning. For even stronger hydrophilic interactions, namely $\Delta\varepsilon_{LS} = +70\%$ (c) and $\Delta\varepsilon_{LS} = +100\%$ (d), the contact line is no longer arrested by the hydrophilic spots. This trend is consistent with Fig. 7: even in the case of the displace droplet, the hydrophilic spots pin the triple line at $\Delta\varepsilon_{LS} = +12\%$ and $+42\%$, leading to non-negligible hysteresis. Conversely, at higher values of $\Delta\varepsilon_{LS}$, the contact line is no longer arrested and the hysteresis becomes negligible. This indicates that the pinning effect is independent of the initial conditions and does not arise from a specific simulation setup.

9 Hysteresis trend 3 lines different droplet size

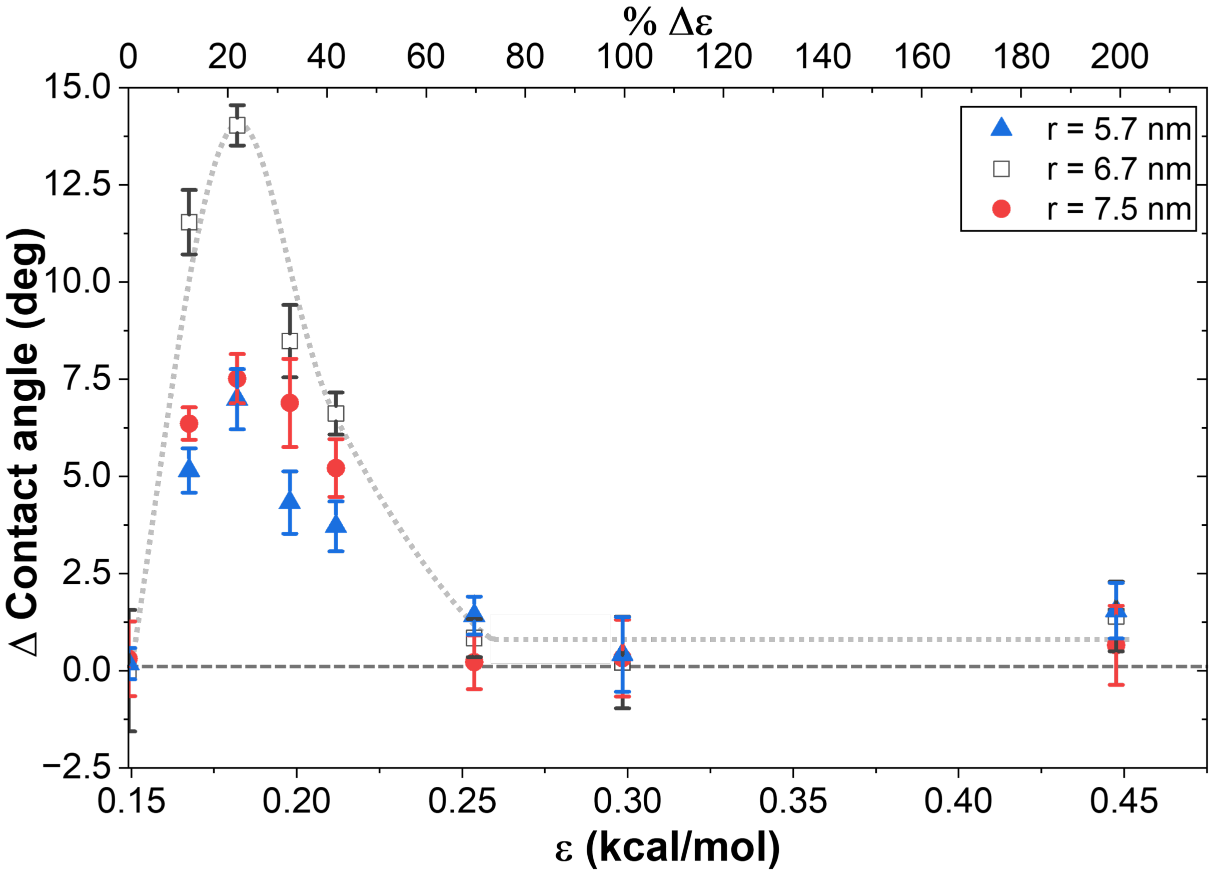

Figure SI-16: Contact-angle hysteresis for the three-line surface for different droplet sizes. The plot shows the absolute value of the difference between the left and right contact angles as a function of the attractive strength $\varepsilon_{LS}^{\alpha}$. Percentage variations on the top axis are given relative to the reference value $\varepsilon_{LS}^{\alpha} = 0.1490$ kcal/mol. The dotted light-gray line reproduces the guideline shown in the corresponding figure in the main text (black open squares). Red circles and blue triangles denote results obtained for droplets that are 50 % larger and 20 % smaller, respectively. Although the absolute values differ among the cases, in the region where the increase in contact angle is observed the corresponding difference between the left and right contact angles remains non-negligible, and the non-monotonic trend with interaction strength is preserved.

10 Absolute value of contact angles in the case of 3 lines hydrophilic spots

	**Contact Angle Max**		**Contact Angle Min**		**Δ Contact Angle**	**Error**
ε (kcal/mol)	Value (°)	Err (°)	Value (°)	Err (°)	(°)	Tot (°)
0.149 20	98.89	1.56	98.89	–	0.00	1.56
0.167 47	113.05	0.21	101.50	0.62	11.54	0.83
0.182 02	111.04	0.31	97.01	0.21	14.03	0.52
0.197 92	109.86	0.38	101.38	0.55	8.48	0.93
0.211 86	113.03	0.32	106.41	0.22	6.62	0.54
0.253 64	103.90	0.26	103.06	0.23	0.84	0.49
0.298 40	101.01	0.25	100.80	0.93	0.21	1.18
0.447 60	96.34	0.73	94.94	0.17	1.40	0.90

Table SI-1: Absolute value of contact angles at the two ends of the cylindrical droplet, together with associated error, hysteresis ($\Delta\Theta = \Theta_{max} - \Theta_{min}$) and associated error.
